# PDHB-AS suppresses cervical cancer progression and cisplatin resistance via inhibition on Wnt/β-catenin pathway

**DOI:** 10.1038/s41419-022-05547-5

**Published:** 2023-02-07

**Authors:** Chi Chi, Wenjie Hou, Yi Zhang, Jie Chen, Zongji Shen, Youguo Chen, Min Li

**Affiliations:** 1 Department of Obstetrics and Gynecology, The First Affiliated Hospital of Soochow University, No. 188 Shizi Road, Suzhou, 215006 Jiangsu China; 2 Department of Obstetrics and Gynecology, Dushu Lake Hospital Affiliated to Soochow University, 9#, Chongwen Rd. SIP, Suzhou, 215000 Jiangsu China

**Keywords:** Cell biology, Cervical cancer

## Abstract

Cervical cancer (CC) is the most prevalent gynecological malignancy occurring in the cervix. Long non-coding RNAs (lncRNAs) can act as oncogenes or anti-oncogenes in CC development. Here, we investigated the functional role and detailed mechanism of lncRNA pyruvate dehydrogenase E1 subunit beta antisense (PDHB-AS) in CC. At first, we found that PDHB-AS was significantly down-regulated in CC cells. Besides, overexpression of PDHB-AS repressed CC cell malignant behaviors. HKF-derived exosomes carried miR-4536-5p to CC cells and thereby inhibited PDHB-AS expression. Moreover, PDHB-AS inactivated the Wnt/β-catenin pathway via impeding the nuclear translocation of β-catenin in CC cells. In addition, miR-582-5p could bind with both PDHB-AS and Dickkopf-1 (DKK1). PDHB-AS recruited poly(A) binding protein cytoplasmic 1 (PABPC1) to inhibit Wnt7b expression. PDHB-AS interacted with RNA-binding motif protein X-linked (RBMX) to regulate cisplatin resistance in CC. Finally, we conducted in vivo experiments to confirm that HKF promoted CC tumor growth whereas PDHB-AS suppressed CC tumor growth. Collectively, PDHB-AS plays a tumor-suppressive role in the progression of CC, which suggests the therapeutic potential of PDHB-AS for CC.

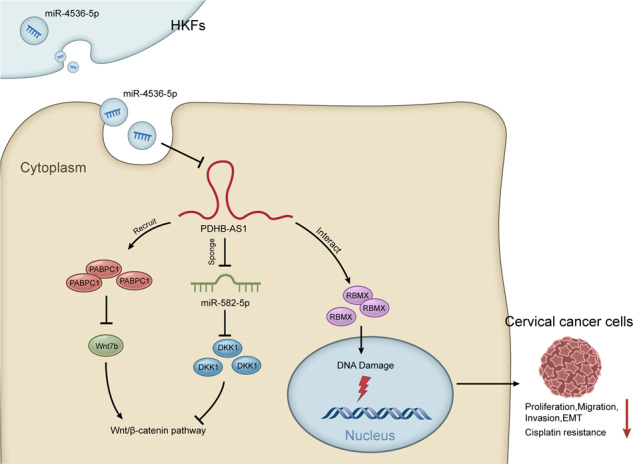

## Introduction

Cervical cancer (CC) has been classified as one of the most predominant malignant cancers in female, becoming a universally concerned public health issue. According to the published report, the incidence and mortality of CC rank the fourth among gynecological cancers worldwide [1]. There are some great advancements in therapeutic approaches including surgery, chemotherapy and radiotherapy [2]. In chemotherapy, cisplatin functions as a key drug for patients with CC at advanced stages. Exosome-transmitted miRNA has been considered as crucial modulators in drug resistance of many cancer cells [3]. However, a considerable number of patients still suffer from pain caused by metastasis and recurrence [4]. Therefore, in-depth exploration into the molecular mechanisms underlying CC development is of great significance for discovery of potential diagnostic biomarkers or therapeutic targets.

Long non-coding RNAs (lncRNAs) belong to ncRNA possessing over 200 nucleotides in length [5]. Most lncRNAs are formed through RNA polymerase II transcription [6]. Accumulating studies have evidenced the involvement of aberrantly expressed lncRNAs in modulating cell differentiation, proliferation, apoptosis, migration, etc. For instance, androgen-stimulated SOCS2-AS1 can strengthen cell proliferative ability and weaken cell apoptotic ability in prostate cancer [7]. Liang et al. have pointed out that PTAF contributes to epithelial-mesenchymal transition (EMT) and invasion-metastasis in serous ovarian cancer [8]. It has been validated that lncRNA-CTS facilitates CC progression via miR-505/ZEB2 axis [9]. LncRNA-TCONS_00026907 has an influence on the progression and prognosis of CC via suppressing miR-143-5p [10]. According to UCSC, we found a novel transcript of lncRNA antisense to pyruvate dehydrogenase E1 subunit beta (PDHB), RP11-802O23.3 (later called PDHB-AS), which locates on chromosome 3 and is 561 bp in length. However, the function and corresponding modulation mechanism of PDHB-AS in CC remain largely unexplored.

Exosomes are extracellular vesicles with endocytic origin, which are secreted by many cells. They can transmit diverse molecules including lncRNAs and miRNAs in microenvironment [11, 12]. Here, we explored the exosome-mediated molecular mechanism in CC progression.

Wnt/β-catenin pathway is widely reported as a crucial modulator in tumor growth and tumor progression. For instance, LINC00210 has been raveled out to stimulate self-renewal and propagation of liver TICs via actuating Wnt/β-catenin pathway [13]. Moreover, NEAT1 can promote the activation of Wnt/β-catenin pathway to exacerbate colorectal cancer development [14]. Nevertheless, the interaction between Wnt/β-catenin pathway and PDHB-AS in CC is still unrevealed.

The aim of the present study is to investigate the functions of PDHB-AS in CC as well as its downstream molecular mechanism, thereby providing new insights into CC treatment.

## Materials and methods

### Cell culture

Human CC cells (MS751, HeLa, C33A, SiHa, and ME-180), human non-cancerous ectocervical epithelial cells (Ect1/E6E7) and Human Embryonic Kidney 293 cells (HEK293) were provided by American Type Culture Collection (ATCC; Manassas, VA, USA). Human keloid fibroblasts (HKFs) were obtained from Huzhen Biotechnology (Shanghai, China). HeLa, C33A, MS751 and SiHa cells were cultured in Eagle’s Minimum Essential Medium (EMEM; Thermo Fisher Scientific, Waltham, MA, USA). ME-180 cells were cultured in McCoy’s 5a Medium (Thermo Fisher Scientific). Ect1/E6E7 cells were maintained in Keratinocyte-Serum Free medium procured from Gibco (Gaithersburg, MD, USA). HKF and HEK293 cells were cultured in DMEM medium (Thermo Fisher Scientific). All culture mediums were supplemented with 10% fetal bovine serum (FBS; Gibco) and replaced every 3 days. All cells were maintained in an incubator which was set as 37 °C and 5% CO_2_. In addition to regular mycoplasma contamination checking, STR analysis was also applied for the authentication of all cells.

### Cell transfection

The pcDNA3.1-PDHB-AS and the empty vector, along with specific siRNAs against PABPC1 (si-PABPC1) or RBMX (si-RBMX) and the corresponding negative control (si-NC), were generated by Genechem (Shanghai, China). Simultaneously, miR-4536-5p mimics, miR-582-5p mimics, and corresponding NC mimics were all obtained from GenePharma (Shanghai, China). Each of these plasmids was transfected into CC cells or HEK293 with the application of Lipofectamine 3000 (Invitrogen, Carlsbad, CA, USA).

### Total RNA extraction and quantitative reverse transcription polymerase chain reaction (RT-qPCR)

RT-qPCR was performed to measure RNA expression as previously described [15]. Total RNA was extracted from CC cells with utilization of Trizol reagent (Invitrogen). RNA concentration and OD260/280 ratios were analyzed with the help of an UV spectrophotometer. Then, 1 μg of RNA was subjected to reverse transcription into cDNA with the application of PrimeScriptRT Reagent Kit procured from TaKaRa (Dalian, China). RT-qPCR reactions were completed with ABI 7300 System (Applied Biosystems, Foster City, CA, USA) along with SYBR Green PCR Master Mix (Takara, Otsu, Shiga, Japan). The thermocycling conditions were as follows: 95 °C for 30 s, followed by 40 cycles of denaturation at 95 °C for 5 s, annealing at 60 °C for 30 s, and extension at 72 °C for 30 s. The relative expression of genes was measured with the 2^−ΔΔCt^ method and was normalized to the internal controls (β-actin or U6). The assay was independently carried out in triplicate.

### Cell counting kit-8 (CCK-8)

This assay was performed in accordance with the procedures described in a previous study [16]. HeLa or SiHa cells were collected at 24 h, 48 h, and 72 h after transfection. Then, 10 μl cell counting kit-8 kit (Dojindo, Kumamoto, Japan) was used to incubate cells for 1 h. Finally, a microplate reader (Molecular Devices, San Jose, CA, USA) was used for measurement of absorbance at 450 nm. The experiment was carried out in triplicate.

### Transwell assay

As described in a previous work [17], 1 × 10^5^ cells were seeded into the upper chamber, which was then inserted into the transwell apparatus (Corning Incorporated, Corning, NY, USA). The upper chamber was pre-coated with Matrigel (BD Biosciences) for detecting cell invasion. The upper chamber pre-coated without Matrigel was used for the analysis of cell migration. Complete medium was added into the lower chamber. Twenty-four hours later, cells which migrated or invaded into the lower chamber were stained using 0.5% crystal violet. Microscope (Nikon, Fukasawa, Japan) was used for observation. The assay was independently carried out in triplicate.

### Western blot

Total protein samples were extracted with RIPA lysis buffer, and then 50 μg of protein was separated on 10% SDS-PAGE, followed by shifting to PVDF membranes. After being blocked with 5% skimmed milk, membranes were incubated with the diluted primary antibodies, including the internal control anti-β-actin (1 µg/ml; Abcam, Cambridge, MA, USA), anti-N-cadherin (1:1000; Cell Signaling Technology, Danvers, MA, USA), anti-E-cadherin (1:1000; Cell Signaling Technology), anti-β-catenin (1:5000; Abcam), anti-CD63 (0.5 µg/ml; Abcam), anti-TSG101 (1:1000; Abcam), anti-Alix (1:1000; Abcam), anti-Wnt7b (1 µg/ml; Abcam), anti-DKK1 (1:1000; Cell Signaling Technology), anti-PABPC1 (0.2 µg/ml; Abcam), anti-RBMX (0.04 µg/ml; Abcam), anti-c-Myc and anti-Cyclin D1 overnight, followed by TBST washing and the incubation with HRP-labeled secondary antibody. The protein bands were finally visualized with ECL Prime Western Blotting Detection reagent (GE Healthcare, Chicago, IL, USA). The intensities of indicated protein bands were calculated by making densitometric analysis of the blots with ImageJ software as previously described [18]. The experiment was carried out in triplicate. The original western blots have been provided in Supplemental Material files.

### Exosome isolation

Exosome isolation was conducted as previously described [19]. After cultured for three days, cell supernatants were collected and centrifuged for removal of cell debris. ExoQuick Plasma prep and Exosome precipitation kit (System Biosciences, Palo Alto, CA, USA) was applied for exosome extraction. For exosomal RNA and protein extraction, exosomes were pretreated with RNase or Proteinase K, respectively. The protein concentration in exosomes was examined by Pierce® BCA Protein Assay kit (Thermo Scientific).

### Nanoparticle tracking analysis (NTA)

Exosome quantification was completed via Nanosight Nanoparticle Tracking Analysis (NTA) as previously described [20]. The size of exosome derived from CC cells was measured with NTA with the application of NanoSight LM10 instrument produced from Malvern Instruments (Westborough, MA, USA.) and equipped with Viton sample room and laser (640 nm). After re-suspension, exosomes were treated with Milli-Q dilution for 500 times and then injected into sample room with the application of sterile syringe. The evaluation of granularity value was conducted using the NTA software, which was in line with the arithmetic value of all particle sizes analyzed by software.

### Transmission electron microscopy (TEM)

TEM was used for exosome characterization as previously described [21]. To characterize the HKF-secreted exosome in CC cells, exosomes (30 μL) were stained with 30 μL of phosphotungstic acid solution (pH = 6.8) and imaged by TEM.

### Exosome labeling

According to the manufacturers’ protocol, 1 μM of PKH26 (Sigma-Aldrich) was used for exosome labeling. Based on previous literature [22], the labeled HKF-secreted exosome were co-cultured with CC cells for 6 h. After 20–30 min fixation in paraformaldehyde and rinse with PBS, DAPI solution procured from Beyotime (Shanghai, China) was utilized for the staining of cell nuclei. Finally, slide observation was performed using Laser scanning microscope produced by Carl Zeiss Meditec (Oberkochen, Germany).

### Luciferase reporter assay

Cignal Finder Reporter Array (QIAGEN, Dusseldorf, Germany) was applied for assessment of the activity of different pathways under PDHB-AS overexpression. PDHB-AS cDNA was PCR amplified and inserted into the firefly luciferase plasmids. The activity of signaling pathway was determined by measuring the activity of their downstream transcription factors.

In addition, the DKK1 3’UTR fragment or full length of PDHB-AS covering miR-582-5p wild-type or mutant binding sites was inserted into pmirGLO luciferase reporter vectors (Promega, Madison, WI, USA), which were then co-transfected with plasmids (miR-582-5p mimics or NC mimics) into HEK293T cells. Cells were collected after 48 h transfection. According to a previous study [23], the luciferase activity of each indicated group was measured with Dual-Luciferase Reporter Assay Kit (E1910, Promega). Each experiment was performed in triplicate.

### TOP/FOP Flash assay

This assay was implemented as previously described [24]. TOP/FOP-Flash (Genechem) was co-transfected into CC cells with pcDNA3.1-PDHB-AS or empty vector (pcDNA3.1). After transfection for 48 h, both firefly and Renila luciferase activities were measured. The luciferase activity of TOP/FOP-Flash was normalized to the Renilla luciferase activity. Experiments were performed in triplicate.

### Fluorescence in situ hybridization (FISH) and immunofluorescence (IF) staining

The specific FISH probe of PDHB-AS was generated by RiboBio (Guangzhou, China). After being fixed with 4% paraformaldehyde, cells were then incubated with PDHB-AS probe at 37 °C overnight. After being washed and blocked with 3% BSA, cells were incubated with RBMX antibody (0.04 µg/ml; Abcam). After nuclear staining with DAPI, images were observed with Olympus fluorescence microscope (Tokyo, Japan). The experiment was carried out in triplicate.

### Immunofluorescence (IF)

For determination of β-catenin location, cells were incubated with β-catenin antibody (1:5000; Abcam) and secondary antibodies conjugated with Alexa Fluor® 488 in succession. After nuclear staining with DAPI, images were observed with Olympus fluorescence microscope (Tokyo, Japan). The experiment was carried out in triplicate.

### RNA immunoprecipitation (RIP) assay

Magna RIPTM RNA-Binding Protein Immunoprecipitation Kit (Millipore, Bedford, MA, USA) was applied in RIP assay. Magnetic beads were conjugated with human primary antibodies at room temperature for 1 h. Normal mouse IgG antibody was used as control. RT-qPCR analysis was done for detection of RNA enrichment after immunoprecipitation.

### RNA pull-down assay

Briefly, the biotin-labeled PDHB-AS/PDHB-AS antisense or biotin-labeled miR-582-5p wild-type/mutant-type was first folded in RNA structure buffer (20 mM Tris–Cl [pH 7.0], 0.2 M KCl and 20 mM MgCl_2_) and then maintained in CC whole-cell lysate for 1 h at 4 °C with rotation. Prior to incubation, CC cell lysate was obtained via briefly sonicating 10 million cells in IP buffer (1 ml, 25 mM Tris [pH 7.4], 0.15 M NaCl, 0.5% NP-40, 0.5 mM DTT, and 1 × complete protease inhibitors [Roche, Basel, Switzerland]) containing 100 U/ml RNase Inhibitor (Thermo Fisher Scientific). RNA-protein complexes were precipitated by streptavidin-coupled T1 beads (Dynabeads), washed five times in IP buffer, and eluted in Laemmli buffer. RNAs or proteins pulled down by biotinylated probes were finally purified and analyzed by RT-qPCR or western blot. The experiment was carried out in triplicate.

### Comet assay

Comet assay was conducted to assess DNA damage under indicated conditions. Briefly, CC cells after transfection were reaped and suspended with PBS. Then cells together with low melting point agarose at the ratio of 1:200 were preserved on a slide which had been coated with 1% regular agarose. The slide solidified at 4 °C was fixed with a cold lysis buffer at 4 °C for 50 min. Then, the slides were dried and dipped in electrophoresis solution. After 25 min, electrophoresis was done at 300 mA, 25 V, followed by dyeing with ethidium bromide. The slide was neutralized, washed, and detected with a fluorescent microscope. The experiment was carried out in triplicate.

### In vivo assay

Five–six-week-old nude mice purchased from Beijing Vital River Laboratory Animal Technology Co., Ltd. (China) were used for xenograft models. HeLa cells transfected with pcDNA3.1, pcDNA3.1-PDHB-AS, and HKF were stably separated with trypsin and washed twice with sterilized PBS. Then 3 × 10^6^ cells in 0.2 ml PBS were subcutaneously inoculated in the flanks of the mice. Tumor growth was monitored every 3 days after being apparently observed and the tumor size was detected using calipers. Four weeks after inoculation, the mice were sacrificed. Animal research was supported by the approval of the First Affiliated Hospital of Soochow University. If the nude mice lost more than 20% body weight, along with tumor metastasis, lethargy, or other painful symptoms, consistent with IACUC criteria, they were inhaled of 4% diethyl ether and sacrificed using cervical dislocation. Tumor volume and weight were detected after mice were sacrificed. Tumor tissues in each group were collected for immunohistochemistry (IHC) analysis.

Paraffin-embedded sections of tumor tissues were placed in an incubator maintained at 60 °C for 2 h and then immersed. The slices were hydrated by ethanol and deionized water. And then, the slices were immersed in citrate buffer solution (0.01 mol/L, pH 6.0) and heated at 100 °C for 30 min. After PBS washing, the slices were subjected to the incubation with 0.5% Triton X-100 for half an hour and then stained with the application of the biotin-streptavidin horseradish peroxidase detection system (CAT. PV-9003, China), produced by ZSGB Biotech. Then, the incubation with primary antibodies, including anti-Ki-67, anti-PCNA, anti-E-cadherin, and anti-N-cadherin were performed at 4 °C overnight. All antibodies were obtained from Abcam. The presence of brown chromogen indicates positive immunoreactivity. Images were visualized using a Nikon microscope.

### Statistical analysis

SPSS version 22.0 (SPSS, IL, USA) was used for statistical analysis. Data from experiments at least in triplicate were shown as mean ± standard (SD). Differences between two groups were analyzed by Student’s t-test, while that among multiple groups were compared by Analysis of Variance (ANOVA). The survival rate of CC patients was analyzed by applying Kaplan–Meier method and calculated with log-rank test. *P* value under 0.05 was regarded as statistically significant.

## Results

### PDHB-AS is down-regulated in CC cells and acts as a tumor suppressor

**Fig. 1:**
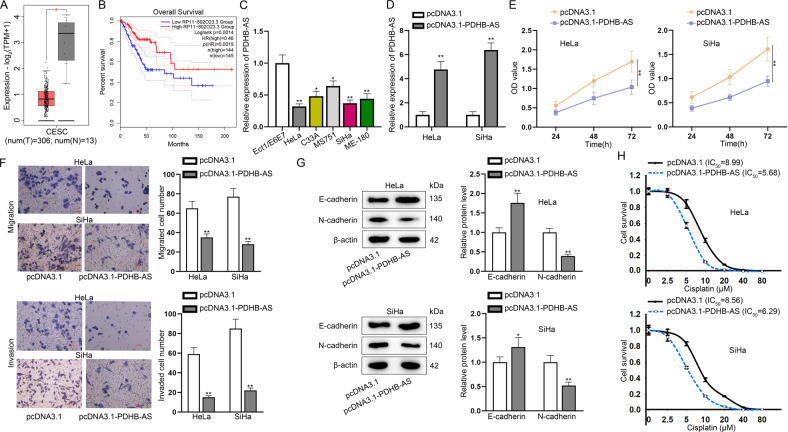
PDHB-AS is down-regulated in CC cells. **A** GEPIA predicted the expression of PDHB-AS in CESC and normal tissues. **B** The association between PDHB-AS expression and overall survival of CESC patients was predicted on GEPIA. **C** PDHB-AS expression levels were examined via RT-qPCR in CC cells (HeLa, C33A, MS751, SiHa and ME-180) versus the normal cell (Ect1/E6E7). **D** PDHB-AS overexpression efficiency was analyzed via RT-qPCR. **E** CCK-8 assay evaluated the proliferation of HeLa and SiHa cells under PDHB-AS overexpression. **F** Transwell assay tested the invasion and migration ability of HeLa and SiHa cells with PDHB-AS overexpression. **G** The protein levels of E-cadherin and N-cadherin in HeLa and SiHa cells were measured by western blot with PDHB-AS overexpression. β-actin was the loading control. **H** The effect of PDHB-AS overexpression on IC_50_ values to cisplatin of HeLa and SiHa cells was measured via CCK-8 assay. *P < 0.05, **P < 0.01.

As demonstrated in GEPIA (http://gepia2.cancer-pku.cn/) database, PDHB-AS was remarkably down-regulated (*P* < 0.05) in 306 specimens of cervical squamous cell carcinoma and endocervical adenocarcinoma (CESC) in relative to 13 normal tissues (Fig. 1A). To confirm the clinical significance of PDHB-AS in CC, we also applied GEPIA database to find the correlation between PDHB-AS expression and overall survival of CC patients. The overall survival rate of patients with higher PDHB-AS expression was higher than those with lower PDHB-AS expression (Fig. 1B). To further verify the role of PDHB-AS in CC, PDHB-AS expression in CC cells was examined with RT-qPCR. The data indicated that PDHB-AS was down-regulated in five CC cell lines in comparison with normal cervical cells (Ect1/E6E7), and the lowest level of PDHB-AS was detected in HeLa and SiHa cells (Fig. 1C; *P* < 0.05, *P* < 0.01). To investigate the impacts of PDHB-AS on the functions of CC cells, gain-of-functional assays were carried out. The overexpression efficiency of pcDNA3.1-PDHB-AS (*P* < 0.01) in HeLa and SiHa cells was confirmed via RT-qPCR analysis (Fig. 1D). Next, it was observed that CC cell proliferation ability was attenuated (*P* < 0.01) by PDHB-AS up-regulation (Fig. 1E). Likewise, based on transwell invasion and migration assays, an overt decline in migrated and invaded cells (*P* < 0.01) was discovered after PDHB-AS overexpression (Fig. 1F). Considering that the EMT process is implicated in cancer invasion and metastasis [25], protein levels of EMT markers were further examined with western blot. The data indicated that PDHB-AS overexpression could contribute to augmented E-cadherin level and lessened N-cadherin level (*P* < 0.01), suggesting that overexpression of PDHB-AS reversed the EMT process of CC cells (Fig. 1G). In addition, we also evaluated the effects of PDHB-AS overexpression on cisplatin resistance of HeLa and SiHa cells. In comparison with the control group, PDHB-AS overexpression resulted in reduction of IC_50_ value to cisplatin (Fig. 1H). In conclusion, PDHB-AS was down-regulated in CC cells, and PDHB-AS overexpression restricted the malignant phenotypes of CC cells.

### HKF-derived exosomal miR-4536-5p down-regulates PDHB-AS in CC cells

**Fig. 2:**
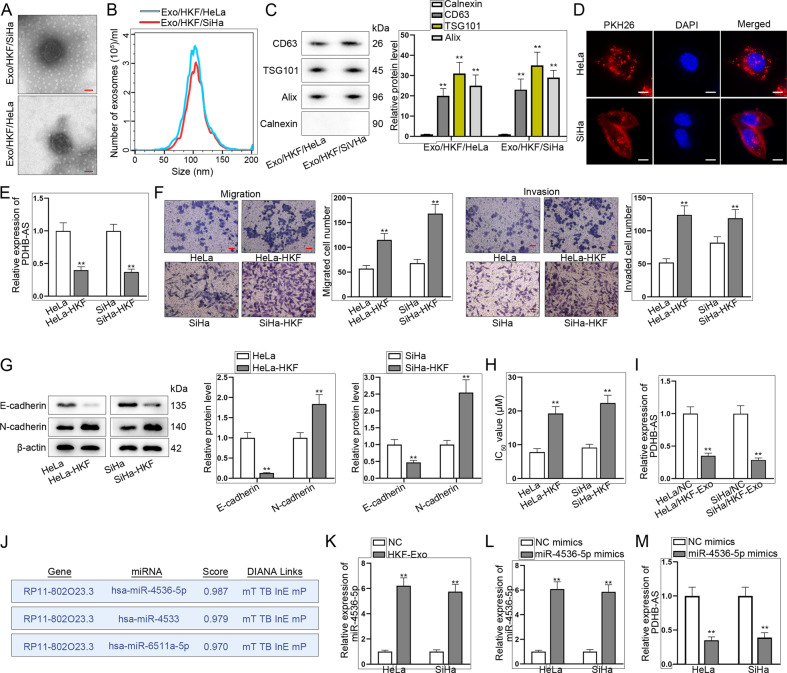
HKF-derived exosomes carry miR-4536-5p and enter CC cells to interfere PDHB-AS expression. **A** Images of exosomes were observed with an electron microscope. **B** Exosome size was analyzed with NTA. **C** Western blot was used to detect exosome marker proteins. **D** PKH26 was used to label and track exosomes. **E** PDHB-AS expression levels in HeLa and SiHa cells versus co-cultured HeLa-HKF and SiHa-HKF were determined by RT-qPCR. **F** Transwell assay tested the invasion and migration ability of cells in different groups. **G** EMT of HeLa and SiHa cells with or without co-culturing with HKFs was determined by detecting EMT-related proteins via western blot. **H** IC_50_ values to cisplatin in several groups were analyzed by CCK-8 assay. **I** PDHB-AS expression levels in HeLa and SiHa cells versus HeLa-HKF/Exo and SiHa-HKF/Exo were determined by RT-qPCR. **J** DIANA was searched to screen out miRNAs which combined with PDHB-AS. **K** miR-4536-5p expression in HeLa and SiHa cells co-cultured with Exo/HKF was detected by RT-qPCR. **L** RT-qPCR examined miR-4536-5p overexpression efficiency in HeLa and SiHa cells. **M** The effect of miR-4536-5p mimics on PDHB-AS expression was examined by RT-qPCR. **P < 0.01.

Next, we investigated the potential mechanism of PDHB-AS in CC cells. HKFs are spindle-shaped which were adherent in culture. Exosomes are nanometric membrane vesicles that could be secreted by almost all kinds of cells, exerting significant importance in intercellular communication [26]. Herein, we speculated HKF-secreted exosomes could be transmitted into CC cells to downregulate PDHB-AS. To prove this hypothesis, we isolated exosomes from CC cells after co-culturing with HKFs. As shown in Fig. 2A, we observed the derived exosomes under an electron microscope. Based on results of NTA and TEM analyses, the exosomes were about 120 nm in diameter and displayed typical cup-shaped morphology, which was in line with the basic morphology of exosomes (Fig. 2B). In addition, the levels of exosome markers (CD63, TSG101, and Alix) further confirmed the presence of exosomes (Fig. 2C, *P* < 0.01). To determine whether HKF-derived exosomes could be transmitted into CC cells, we labeled purified HKF-derived exosomes with the red fluorescent lipid dye PKH26 for exosomes tracking. After co-culturing of receptor cells (HeLa and SiHa) with PKH26-labeled exosomes, red fluorescence-positive puncta were observed in receptor cells, mainly in cell cytoplasm (Fig. 2D). Next, PDHB-AS expression was detected by RT-qPCR, and it was found that the level of PDHB-AS in CC cells was overtly lower after co-culturing with HKFs (*P* < 0.01) than that in independently cultured CC cells (Fig. 2E). In addition, we found that CC cell migration and invasion were promoted (*P* < 0.01) after CC cells were co-cultured with HKFs (Fig. 2F). Meanwhile, western blot results manifested that E-cadherin was decreased while N-cadherin level was increased (*P* < 0.01) in CC cells co-cultured with HKFs (Fig. 2G). Moreover, the IC_50_ value was markedly augmented (*P* < 0.01) after CC cells were co-cultured with HKFs (Fig. 2H). Subsequently, PDHB-AS expression was detected to be much lower (*P* < 0.01) in CC cells treated with HKF-derived exosomes (Fig. 2I). Based on above results, we suspected that HKF-derived exosomes could inhibit PDHB-AS expression in CC cells. We applied DIANA database (http://diana.imis.athena-innovation.gr/DianaTools/index.php) and found that miR-4536-5p was the miRNA with highest possibility to combine with PDHB-AS (Fig. 2J). Then we identified the basal level of miR-4536-5p through measuring its copy number in two CC cells (Figure S1A). Moreover, we detected the copy number and expression of miR-4536-5p in CC cells with or without HKF-derived exosomes co-culturing. It was discovered that miR-4536-5p copy number was increased (*P* < 0.01) in CC cells with Exo/HKF culture (Figure S1B). In addition, the results of RT-qPCR revealed that miR-4536-5p expression was obviously increased (*P* < 0.01) in CC cells treated with HKF-derived exosomes (Fig. 2K). In addition, we overexpressed miR-4536-5p in CC cells (Fig. 2L; *P* < 0.01) and revealed that the transfection of miR-4536-5p mimics also led to a marked decline (*P* < 0.01) in PDHB-AS expression in CC cells (Fig. 2M). Taken together, HKF-derived exosomes transferred miR-4536-5p to CC cells to interfere PDHB-AS expression.

### PDHB-AS inactivates Wnt/β-catenin pathway via regulating Wnt7b and DKK1

**Fig. 3:**
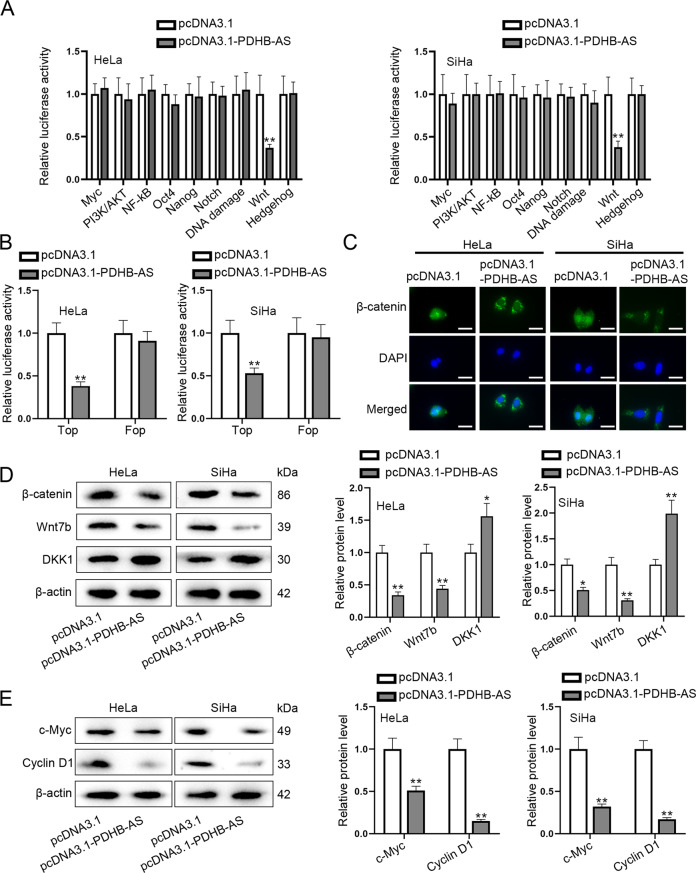
PDHB-AS inactivates the Wnt/β-catenin pathway by regulating Wnt7b and DKK1. **A** Luciferase reporter assay measured the activity of different pathways in HeLa and SiHa cells. **B** The activity of β-catenin was detected through TOP/FOP flash luciferase reporter assay. **C** IF staining detected the localization of β-catenin in CC cells. **D** Western blot analysis was performed to measure the protein levels of Wnt7b, DKK1, and β-catenin under PDHB-AS overexpression. **E** Western blot examined the protein levels of downstream targets of CTNNB1 (c-Myc and Cyclin D1) in CC cells with PDHB-AS overexpression. **P < 0.01.

Considering that signaling pathways are closely associated with cancer development, we conducted luciferase reporter assay to screen out the pathway which might be involved in PDHB-AS regulatory mechanism in CC cells. It was manifested in experimental results that Wnt luciferase activity was weakened (*P* < 0.01) under PDHB-AS overexpression (Fig. 3A). Afterwards, TOP/FOP flash assay was performed to assess the transcription activity of β-catenin in Wnt pathway. As indicated in Fig. 3B, PDHB-AS overexpression obviously repressed the luciferase activity of TOP (*P* < 0.01), and it suggested that PDHB-AS could inactivate the Wnt/β-catenin pathway. Besides, accumulation of β-catenin in the nucleus was obstructed upon PDHB-AS up-regulation (Fig. 3C). It indicated that PDHB-AS overexpression inhibited the nuclear import of β-catenin, thus inactivating Wnt/β-catenin pathway. Moreover, expression of key genes involved in Wnt/β-catenin pathway was detected via RT-qPCR and western blot, and PDHB-AS overexpression was observed to hardly influence CTNNB1 expression but overtly lessen the protein level of β-catenin. Simultaneously, PDHB-AS up-regulation decreased the mRNA and protein levels of Wnt7b while elevating DKK1 levels (Figures S1C, 3D; *P* < 0.01). Similarly, it was suggested in western blot analysis that PDHB-AS overexpression could lead to decline in c-Myc and Cyclin D1 proteins (downstream targets of CTNNB1) (Fig. 3E; *P* < 0.01). All these data indicated that PDHB-AS inactivated the Wnt/β-catenin pathway via regulating Wnt7b and DKK1.

### PDHB-AS competitively binds to miR-582-5p to enhance DKK1 expression

**Fig. 4:**
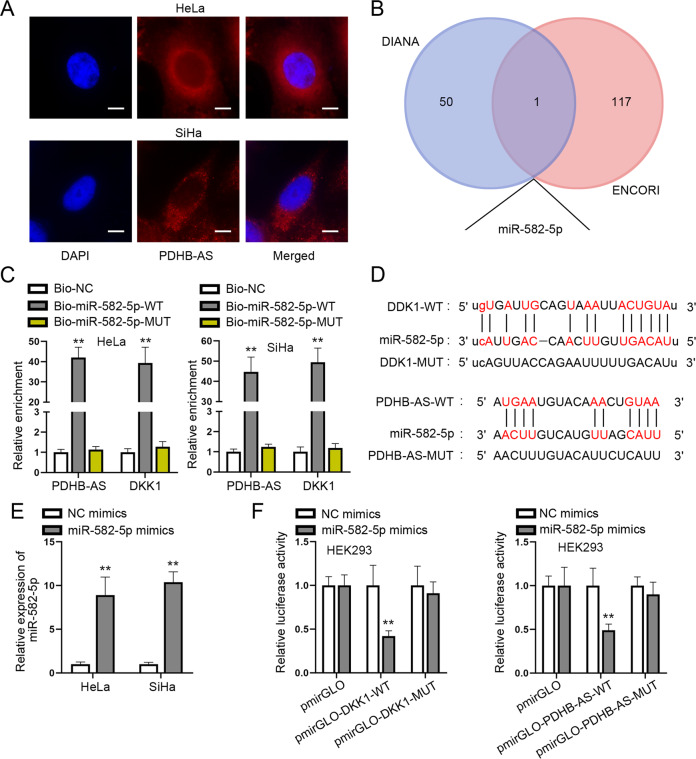
PDHB-AS competitively binds to miR-582-5p to enhance DKK1 expression. **A** FISH assay detected the distribution of PDHB-AS in CC cells. **B** miR-582-5p that might bind with PDHB-AS and DDK1 was predicted via starBase and DIANA. **C** RNA pull down assays detected the enrichment of PDHB-AS and DKK1 in Bio-miR-582-5p-WT/MUT. **D** The binding sites of miR-582-5p on DKK1 and PDHB-AS were predicted severally by DIANA and starBase. **E** RT-qPCR detected miR-582-5p expression in HeLa and SiHa cells after miR-582-5p overexpression. **F** The luciferase activity of indicated groups was measured for further confirmation of the affinity between miR-582-5p and DKK1/PDHB-AS. **P < 0.01.

To unveil the molecular mechanism of PDHB-AS in regulating the Wnt/β-catenin pathway in CC cells, we conducted FISH assay to determine the subcellular distribution of PDHB-AS in HeLa and SiHa cells. PDHB-AS was verified to be mainly located in the cytoplasm, which indicated that PDHB-AS participated in post-transcriptional regulation to exert functions (Fig. 4A). Through starBase v2.0 (https://starbase.sysu.edu.cn/) and DIANA tools, only 1 miRNA (hsa-miR-582-5p) was predicted to has the potential to bind to both PDHB-AS and DKK1 (Fig. 4B). Then as shown in RNA pull down assay results, both PDHB-AS and DKK1 were highly precipitated (*P* < 0.01) in Bio-miR-582-5p-WT rather than Bio-miR-582-5p-MUT and Bio-NC groups (Fig. 4C). Besides, miR-582-5p binding sites on PDHB-AS and DKK1 predicted on starBase were displayed, as well as the corresponding mutated sites (Fig. 4D). The transfection of miR-582-5p mimics resulted in a significant augment (*P* < 0.01) of miR-582-5p expression (Fig. 4E) and then luciferase reporter experiments were done to verify the effectiveness of miR-582-5p binding sites on PDHB-AS and DKK1. The data revealed that miR-582-5p overexpression led to a reduction (*P* < 0.01) in the luciferase activity of pmirGLO-DKK1-WT and pmirGLO-PDHB-AS-WT, while barely affecting that of pmirGLO-DKK1-MUT and pmirGLO-PDHB-AS-MUT (Fig. 4F). To identify whether PDHB-AS and miR-582-5p could regulate each other, we applied RT-qPCR to analyze the expression changes of them responding to their respective overexpression. It was uncovered that PDHB-AS and miR-582-5p had no regulatory relation in regard to expression level (Figure S2B). To verify the effect of miR-582-5p on the role of PDHB-AS in CC cells, a series of rescue functional assays were carried out. Results of CCK-8 assay showed that overexpressed miR-582-5p could offset the inhibitory impact of PDHB-AS up-regulation on cell viability (Figure S3A; *P* < 0.01). Based on transwell assays, suppressed cell migratory and invasive abilities induced by elevated PDHB-AS were recovered by miR-582-5p augment (Figure S3B, C; *P* < 0.01). Figure S3D demonstrated that EMT process reversed by the transfection with pcDNA3.1-PDHB-AS was recovered again after co-transfection of miR-582-5p mimics (*P* < 0.01). Moreover, Figure S1C–F manifested PDHB-AS-MUT had no influence on malignant behaviors of CC cells. Finally, rescue assay was implemented to confirm the interaction among PDHB-AS, miR-582-5p, and DKK1. The outcomes revealed PDHB-AS-mediated DKK1 elevation could be restored by overexpressed miR-582-5p (Figure S3E; *P* < 0.01). In summary, PDHB-AS bound to miR-582-5p to enhance DKK1 expression in CC cells and exerted its inhibitory impacts on CC cell biological behaviors via miR-582-5p/DKK1.

### PDHB-AS recruits PABPC1 to modulate Wnt7b expression

**Fig. 5:**
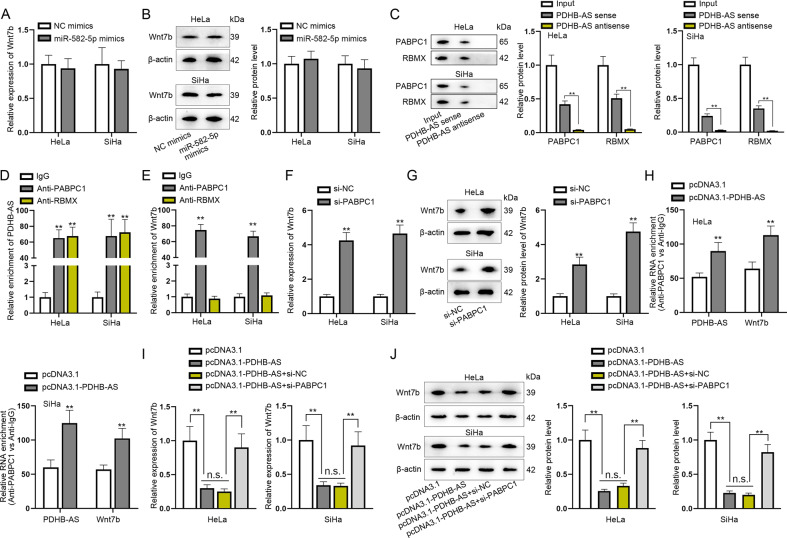
PDHB-AS recruits PABPC1 to modulate Wnt7b expression. **A**, **B** The mRNA and protein levels of Wnt7b were measured respectively by RT-qPCR and western blot after miR-582-5p overexpression. **C** PABPC1 and RBMX were ascertained to bind to PDHB-AS by RNA pull down assays and mass spectrometry, followed by western blot to detect PABPC1 and RBMX protein levels precipitated in PDHB-AS sense/antisense, with input as a positive control. **D**, **E** In RIP assay, the relative enrichment of PDHB-AS and Wnt7b in anti-PABPC1 and anti-RBMX groups was detected via RT-qPCR, with IgG as a negative control. **F**, **G** The mRNA and protein levels of Wnt7b were examined respectively by RT-qPCR and western blot after knockdown of PABPC1. **H** In RIP assay, the relative enrichment of PDHB-AS or Wnt7b precipitated in anti-PABPC1 was measured in cells transfected with pcDNA3.1 or pcDNA3.1-PDHB-AS, respectively. **I**, **J** RT-qPCR and western blot analyses of Wnt7b levels were performed in HeLa and SiHa cells transfected with pcDNA3.1, pcDNA3.1-PDHB-AS, pcDNA3.1-PDHB-AS + si-NC, or pcDNA3.1-PDHB-AS + si-PABPC1. **P < 0.01, n.s.: no significance.

Based on above results, we hypothesized PDHB-AS might modulate Wnt7b via miR-582-5p. Thus, Wnt7b was detected in miR-582-5p overexpressed cells and it was suggested from the experimental results that miR-582-5p overexpression could not regulate the mRNA and protein levels of Wnt7b (Fig. 5A, B). For this reason, we guessed whether PDHB-AS interacted with a certain protein to regulate Wnt7b. Therefore, we performed RNA pull down assays to find the potential protein interacting with PDHB-AS. Proteins of CC cells were incubated and pulled down by biotin-labeled PDHB-AS. Mass spectrometry analyses were performed to analyze the purified proteins. As shown in Fig. 5C, PABPC1 and RBMX were specifically identified (*P* < 0.01). To further verify the interaction between PABPC1 and PDHB-AS and between RBMX and PDHB-AS, we performed RIP experiments, and the results showed that PDHB-AS was highly precipitated (*P* < 0.01) in Anti-PABPC1 and Anti-RBMX (Fig. 5D). Meanwhile, it was suggested in RIP results that Wnt7b was largely abundant (*P* < 0.01) in Anti-PABPC1 rather than in Anti-RBMX (Fig. 5E). To determine whether Wnt7b expression was regulated by PABPC1, CC cells were transfected with si-PABPC1, and it was found both Wnt7b mRNA and protein levels were increased (*P* < 0.01) by si-PABPC1 (Fig. 5F, G). From these results, we verified the binding and negative correlation between PABPC1 and Wnt7b. Later, we found that overexpressed PDHB-AS could strengthen the combination of PABPC1 and PDHB-AS or PABPC1 and Wnt7b (Fig. 5H; *P* < 0.01). Notably, down-regulated PABPC1 was discovered to countervail the inhibiting effects of PDHB-AS overexpression on Wnt7b mRNA and protein levels (Fig. 5I, J; *P* < 0.01, n.s.: no significance). In conclusion, PDHB-AS recruited PABPC1 to regulate Wnt7b expression in CC cells.

### PDHB-AS interacts with RBMX to affect cisplatin resistance in CC cells

**Fig. 6:**
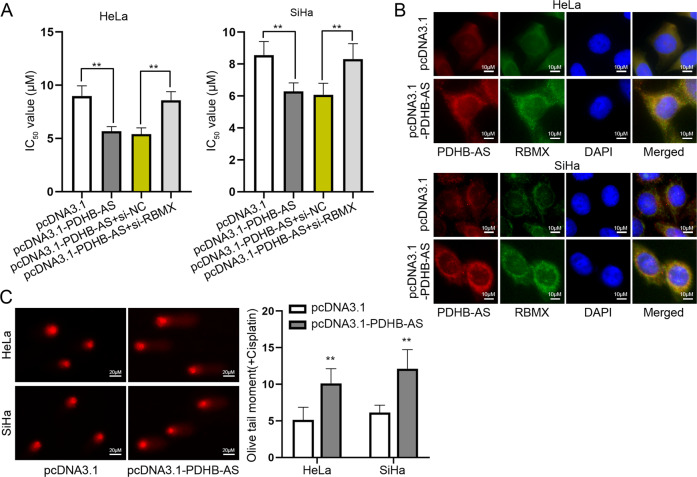
PDHB-AS interacts with RBMX to affect cisplatin resistance in CC cells. **A** IC_50_ values to cisplatin in HeLa and SiHa cells transfected with indicated plasmids including pcDNA3.1, pcDNA3.1-PDHB-AS, pcDNA3.1-PDHB-AS + si-NC, or pcDNA3.1-PDHB-AS + si-RBMX were detected. **B** IF and FISH experiments detected the distribution of PDHB-AS and RBMX in HeLa and SiHa cells with PDHB-AS overexpression. **C** DNA damage in HeLa and SiHa cells transfected with pcDNA3.1-PDHB-AS upon cisplatin treatment was detected by comet assays. **P < 0.01.

Based on above-mentioned results, RBMX was the protein interacting with PDHB-AS. To assess the role of RBMX in CC cells, we conducted functional assays in RBMX-silenced cells. Through CCK-8 assays, RBMX deletion abrogated the suppressing effects of PDHB-AS up-regulation on IC_50_ value to cisplatin (Fig. 6A; *P* < 0.01). Besides, we demonstrated that PDHB-AS and RBMX co-existed in cell cytoplasm by IF and FISH assays (Fig. 6B). In addition, we found that PDHB-AS facilitated the DNA damage, as indicated by comet assay results that the olive tail moment was longer when PDHB-AS was overexpressed (Fig. 6C; *P* < 0.01). Collectively, PDHB-AS interacted with RBMX to affect DNA damage and cisplatin resistance in CC cells.

### PDHB-AS suppresses the tumorigenesis of CC in vivo

**Fig. 7:**
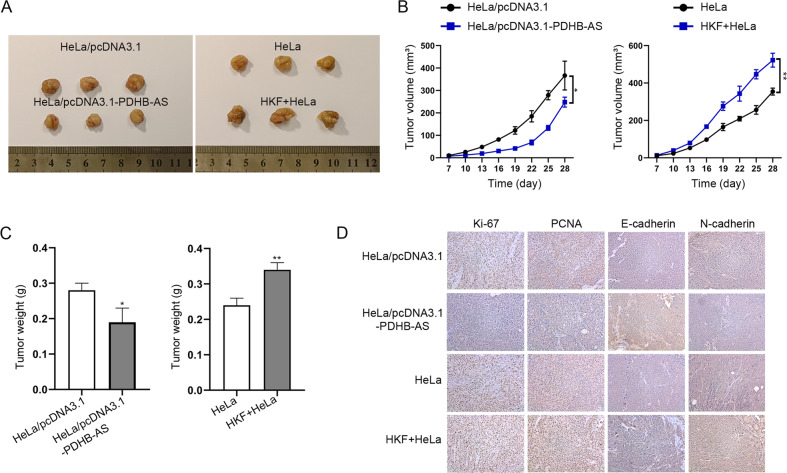
PDHB-AS suppresses the tumorigenesis of CC in vivo. Mice were injected with HeLa cells with pcDNA3.1 or pcDNA3.1-PDHB-AS transfection or HeLa cells with or without HKF co-cultivation, and then in vivo assays were carried out. **A** Tumors in different groups were resected and displayed. **B**, **C** Tumor volumes and weights were measured under different conditions. **D** The levels of proliferation markers (Ki-67 and PCNA) as well as EMT markers (E-cadherin and N-cadherin) were detected in different groups of tissues. *P < 0.05, **P < 0.01.

In order to testify the modulatory function of HKF and PDHB-AS on CC tumorigenesis, in vivo assays were performed. The results indicated that PDHB-AS overexpression resulted in a remarkable reduction in tumor volume and weight, while HKF exerted opposite effects (*P* < 0.05, *P* < 0.01) (Fig. 7A–C). The proliferation and EMT markers were also detected in tumor tissues collected from different groups of mice. As illustrated in Fig. 7D, the levels of Ki-67, PCNA, and N-cadherin in PDHB-AS overexpression group were lower than control group, while the level of E-cadherin was higher in PDHB-AS overexpression group. The levels of these markers were changed in an opposite tendency when treated with HKF. Briefly, PDHB-AS suppressed the tumorigenesis of CC in vivo.

## Discussion

In the current research, we first confirmed that PDHB-AS was down-regulated in CC cells and overexpression of PDHB-AS inhibited tumor growth and cisplatin resistance in CC. Functionally, miR-4536-5p transferred from HKF-secreted exosomes inhibited PDHB-AS expression. PDHB-AS interacted with PABPC1 to downregulate Wnt7b and absorbed miR-582-5p to up-regulate DKK1, which impeded the nuclear import of β-catenin and inactivated Wnt/β-catenin pathway. In addition, PDHB-AS interacted with RBMX to elevate the DNA damage in CC cells. Taken together, this data sustained our conclusion that PDHB-AS exerted tumor-suppressive function in CC.

Recent researches have revealed that exosomes facilitate tumor progression and enhance drug resistance in cancer cells by delivering oncogenic DNA, protein, and ncRNA [27]. Exosomes contain specific repertoires of ncRNAs, including miRNA and lncRNA, indicating that a specific RNA sorting mechanism may exist [28]. Recent reports show that HKF-derived exosomes function as tumor suppressors or oncogenes in various cancers and affect chemoresistance [29, 30], which has not been extensively assessed in CC. In this study, we proved that HKF-derived exosomes carried miR-4536-5p to CC cells to inhibit PDHB-AS. Our data indicated that the cisplatin resistance was significantly enhanced in CC cells co-cultured with HKF. Moreover, the tumor growth was also facilitated in CC cells co-cultured with HKF in vivo. Nonetheless, further in vivo experiments should be performed to completely explore efficacy of combined treatments with PDHB-AS.

Although miR-4536-5p negatively modulated PDHB-AS expression to some extent, it could not completely prevent PDHB-AS from continuing to play its tumor suppressing functions. Recently, the study on CC has evolved from the cell level to the molecular level, from the gene level to the protein level [31]. Moreover, Wnt/β-catenin pathway is a key signaling cascade participating in reproductive system organogenesis and various oncogenesis. Disturbance of the Wnt/β-catenin signaling pathway is related to a high incidence of cancers [32–34]. In our study, we found that PDHB-AS overexpression could inactivate the Wnt/β-catenin signaling pathway to inhibit the proliferation and EMT process by regulating DKK1 and Wnt7b.

It is well known that lncRNAs can modulate gene expression through different methods. One of the most important methods is to antagonize the function of miRNAs by competing with mRNAs to bind to common target miRNAs [35]. For example, lncRNA TDRG1 targets miR-326 to regulate MAPK1 expression, and thereby promoting CC cell proliferation, migration, and invasion [36–38]. In our research, bioinformatics analysis and luciferase reporter assay showed that PDHB-AS competitively bound to miR-582-5p to enhance DKK1 expression in CC cells. Lee et al. have reported that the stable expression of DKK1 blocks the nuclear translocation of β-catenin, resulting in down-regulation of its downstream targets (VEGF and cylcin D), whereas knockdown of DKK1 abrogates this blocking [39]. On the contrary, miR-92a promotes cell viability and invasion in CC, partly at least, via inhibiting the protein expression of DKK1 [40]. As expected, our studies further showed that PDHB-AS inactivated the Wnt/β-catenin pathway by up-regulating DKK1 in CC cells.

Despite investigations of miRNA and lncRNA interactions, lncRNA binding protein activity is not well researched in the context of post-transcriptional regulation. In this study, we revealed that PDHB-AS could bind to PABPC1 to knock down Wnt7b. It has been unveiled that Wnt7b can serve as a primary determinant of differential Wnt/β-catenin activation in pancreatic adenocarcinoma (PDAC) [41]. Likewise, PDHB-AS disrupted the interaction between Wnt/β-catenin ligands and their receptors, which may be a particularly therapeutic approach for CC. RBMX accumulates at DNA lesions through multiple domains in a poly (ADP-ribose) polymerase 1-dependent manner and promoted homologous recombination (HR) through elevating BRCA2 expression [42]. RBMX belongs to a small protein family with additional members encoded by paralogs on the mammalian Y chromosome and other chromosomes. These RNA-binding proteins are crucial for normal development, and are also associated with cancer progression and viral infection [43, 44]. Most interestingly, we found that PDHB-AS combined with RBMX to inhibit cisplatin resistance in CC cells.

## Conclusion

Conclusively, our study elucidated the functional role of PDHB-AS in CC, which might be a novel target for CC treatment.

## Supplementary information

Supplemental materials

Reproducibility Checklist

C33A

Ect1 E6E7

HEK293T

HeLa

ME-180

MS751

SiHa

Original Data File

Authorship change file

## Data Availability

The data underlying this article will be shared on reasonable request to the corresponding author.
