# Supplementary material for: PDHB-AS suppresses cervical cancer progression and cisplatin resistance via inhibition on Wnt/β-catenin pathway
Source: Cell Death Dis. 2023 Feb 7;14(2):90. doi: 10.1038/s41419-022-05547-5 (PMC9905568; doi:10.1038/s41419-022-05547-5)
Supplement: Supplementary file 1 — Supplemental materials [file 41419_2022_5547_MOESM1_ESM.docx]

**Figure S1 Copy number of miR-4536-5p in CC cells**

**
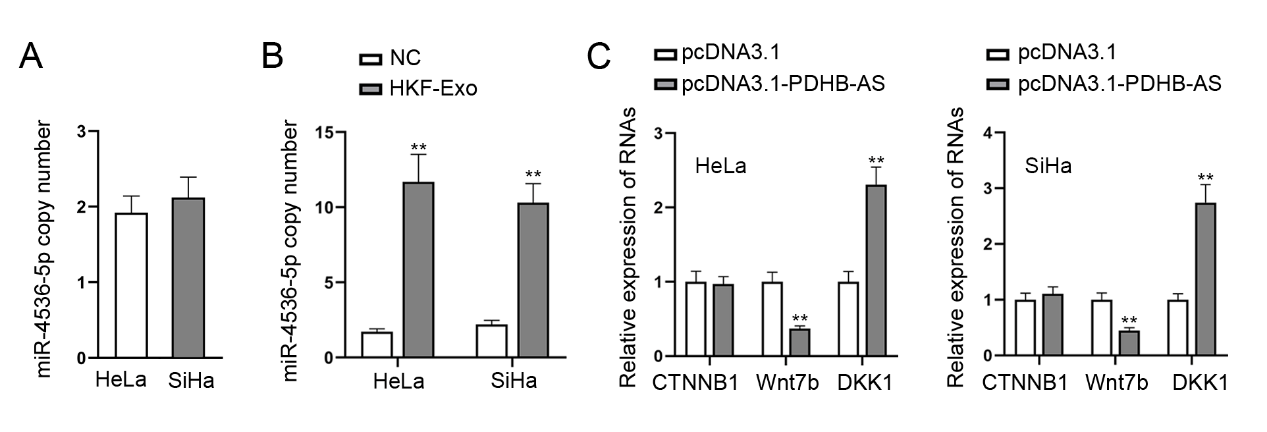
**

(A) Copy number of miR-4536-5p in two CC cells was detected. (B) Copy number of miR-4536-5p in CC cells after co-culturing with Exo/HKF was detected. (C) The mRNA levels of Wnt/β-catenin pathway key genes were measured via RT-qPCR. ^**^P < 0.01.

**Figure S2 PDHB-AS and miR-582-5p cannot regulate each other**

**
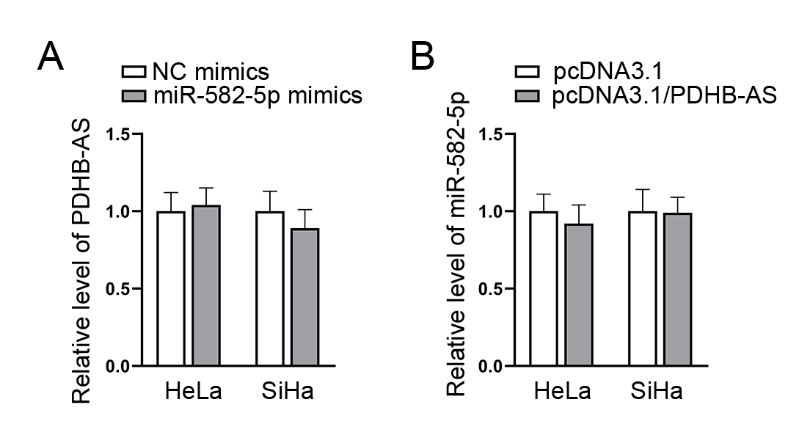
**

(A) The expression level of PDHB-AS in CC cells after miR-582-5p was overexpressed was measured by RT-qPCR. (B) The expression level of miR-582-5p in CC cells with PDHB-AS overexpression was measured by RT-qPCR.

**Figure S3 PDHB-AS suppresses CC progression by interacting with miR-582-5p**

**
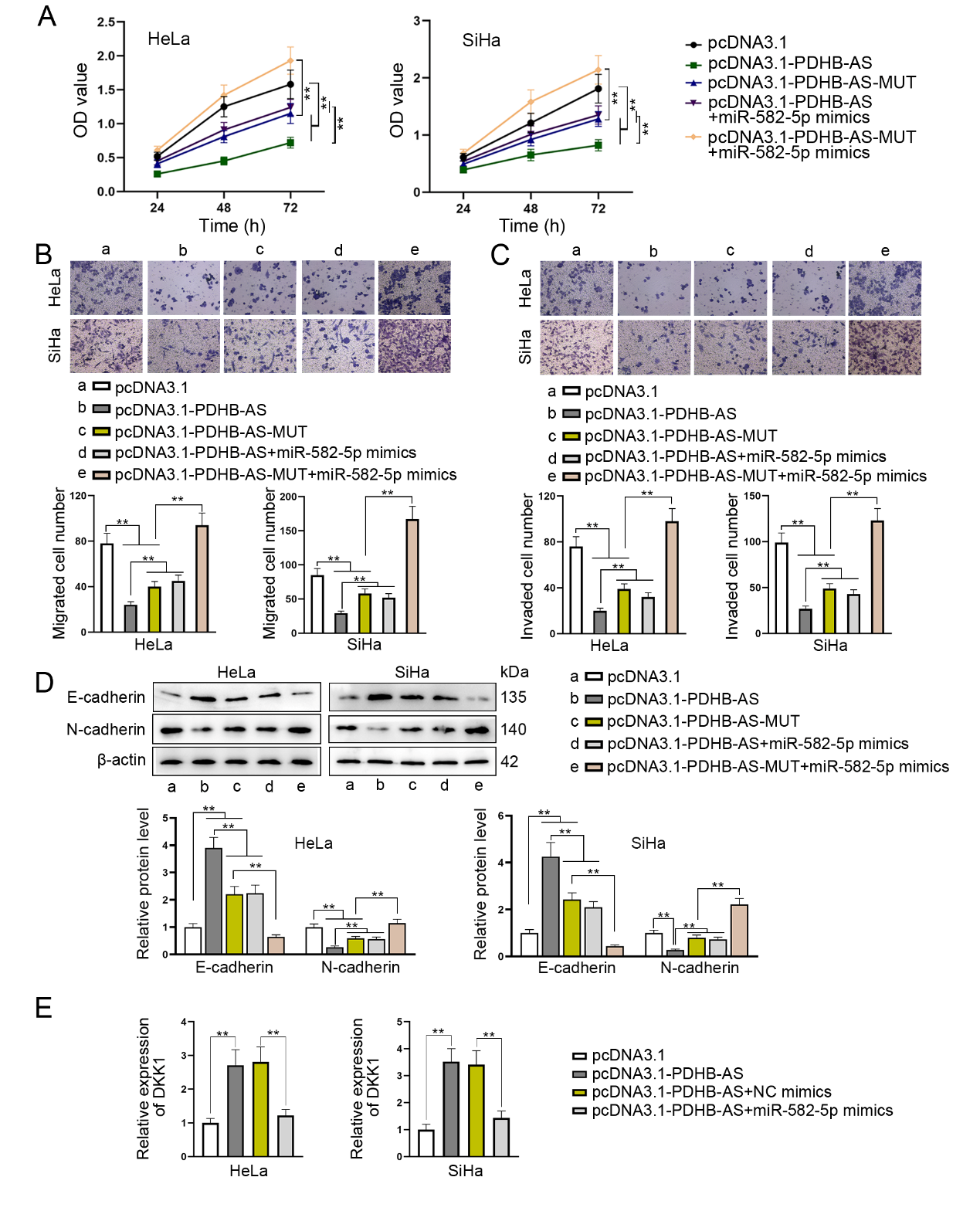
**

(A) CCK-8 assay was conducted to observe cell viability after transfection of indicated plasmids including pcDNA3.1, pcDNA3.1-PDHB-AS, pcDNA3.1-PDHB-AS-MUT, pcDNA3.1-PDHB-AS+miR-582-5p mimics, and pcDNA3.1-PDHB-AS-MUT+miR-582-5p mimics. (B-C) Transwell assays were performed to detect cell migratory and invasive capabilities in several groups. (D) Western blot was used to quantify EMT-related markers in cells transfected with indicated plasmids. (E) DKK1 expression was examined via RT-qPCR in cells with different transfections. ^**^P < 0.01.
