# Supplementary material for: PDHB-AS suppresses cervical cancer progression and cisplatin resistance via inhibition on Wnt/β-catenin pathway
Source: Cell Death Dis. 2023 Feb 7;14(2):90. doi: 10.1038/s41419-022-05547-5 (PMC9905568; doi:10.1038/s41419-022-05547-5)
Supplement: Supplementary file 3 — C33A [file 41419_2022_5547_MOESM3_ESM.pdf]

Cell Line Authentication Service

STR Profiling Report

Sample From: Guangdong Provincial People's Hospital

Sample Type: Cell Line

Testing Method: STR Genotyping

Report Time: 12/24/2021

Add: Room 502, NO.2 Zhongxing Creative Park, Lane 1015, LongtengRoad, Songjiang District, Shanghai/ Tel:+86-021-33559493
E-mail: market@biowing.com

COMPANY STATEMENT

1. THIS REPORT IS ONLY RESPONSIBLE FOR THE SAMPLES ANALYZED.
2. THE TESTING RESULTS AND THE ORGANIZATION NAME WILL NOT BE USED FOR ADVERTISEMENT, COMMERCIAL EXHIBITIONS, COMMERCIAL PERFORMANCE AND OTHER COMMERCIAL ACTIVITIES.
3. OBJECTIONS SHOULD BE RAISED WITHIN FIFTEEN DAYS AFTER THE RECEIPT OF THIS REPORT.
4. THE PAPER REPORT WITH CONTENT ALTERING, ADDING OR WITHOUT THE STAMPED SEAL OF THE COMPANY ARE INVALID.

Testing Company: Shanghai Chenying Biotechnology Co. Ltd
Address: Room 502, NO.2 Zhongxing Creative Park, Lane 1015, LongtengRoad, Songjiang District, Shanghai
Tel: +86-021-33559493
Contact: Wenyao Zhang
E-mail: market@biowing.com

Cell Line Authentication – STR Profiling Report

Sample code

Table 1. Sample Code

Customer's code	Company Code
C33A	20211224-01

Sample Number:1

Sample Type: Cell line

Testing Type: STR

Testing Method:

DNA was extracted by a commercial kit from CORNING (AP-EMN-BL-GDNA-250G). The twenty STRs including Amelogenin locus were amplified by six multiplex PCR and separated on ABI 3730XL Genetic Analyzer. The signals were then analyzed by the software GeneMapper.

Data Interpretation:

Cell lines were authenticated using Short Tandem Repeat (STR) analysis asdescribed in 2012 in ANSI Standard (ASN-0002) by the ATCC Standards Development Organization (SDO) and in Capes-Davis et al., Match criteria for human cell line authentication: Where do we draw the line? Int J Cancer.2013;132(11):2510-9.

Test Results

1. STR profile

Table 2. STR and Amelogenin Genotyping Results of Cell line.

Loci	**Sample information**			**Cell Bank information**		
	Sample name：C33A			Cell line name：C33A		
	**Allele1**	**Allele2**	**Allele3**	**Allele1**	**Allele2**	**Allele3**
D5S818	11	12		11	12	
D13S317	13			13		
D7S820	10			10		
D16S539	13	14		13	14	
VWA	18	20		18	20	
TH01	7	8		7	8	
AMEL	X	X		X	X	
TPOX	9			9		
CSF1PO	12			12		
FGA	21	26				
Penta E	6	8				
Penta D	10					
D21S11	29	30	31			
D18S51	15	17	18			
D8S1179	10	14				
D3S1358	16					
D19S433	11	13	14			
D2S1338	23	25				

Add: Room 502, NO.2 Zhongxing Creative Park, Lane 1015, LongtengRoad, Songjiang District, Shanghai/ Tel:+86-021-33559493
E-mail: market@biowing.com

2. database annotation

Figure 1. STR matching analysis

**Cell line name**	C-33 A
**Synonyms**	C33A; C33a; C33-A; C-33-A; C-33A; C33
**Accession**	CVCL_1094
**Resource Identification Initiative**	To cite this cell line use: C-33 A (RRID:CVCL_1094)
**Comments**	Part of: Cancer Dependency Map project (DepMap) (includes Cancer Cell Line Encyclopedia - CCLE).<br>Part of: COSMIC cell lines project.<br>Part of: PTEN genetic alteration cell panel (ATCC TCP-1030).<br>Doubling time: 1.36 days (PubMed=29156801).<br>Microsatellite instability: Instable (MSI-high) (PubMed=31068700; Sanger).<br>Omics: Deep exome analysis.<br>Omics: Deep RNAseq analysis.<br>Omics: DNA methylation analysis.<br>Omics: Protein expression by reverse-phase protein arrays.<br>Omics: SNP array analysis.<br>Omics: Transcriptome analysis.
**Sequence variations**	Mutation; HGNC; 11998; TP53; Simple; p.Arg273Cys (c.817C>T); ClinVar=VCV000043594; Zygosity=Unspecified (PubMed=1648218).
**Genome ancestry**	**Source:** PubMed=30894373<br>Origin / % genome:<br>African 0.41<br>Native American 0.33<br>East Asian, North 0.48<br>East Asian, South 0.19<br>South Asian 0.82<br>European, North 66.58<br>European, South 31.19
**Disease**	Cervical squamous cell carcinoma, not otherwise specified (NCIt: C4028)<br>Squamous cell carcinoma of the cervix uteri (ORDO: Orphanet_213767)
**Species of origin**	Homo sapiens (Human) (NCBI Taxonomy: 9606)
**Species of origin**	Homo sapiens (Human) (NCBI Taxonomy: 9606)
**Sex of cell**	Female
**Age at sampling**	66Y
**Category**	Cancer cell line
**STR profile**	**Source(s):** AddexBio; ATCC; CCRID; Cosmic-CLP; DepMap; PubMed=25877200<br>**Markers:**<br>Amelogenin: X<br>CSF1PO: 12<br>D2S1338: 23,25<br>D3S1358: 16<br>D5S818: 11,12<br>D7S820: 10<br>D8S1179: 10,14<br>D13S317: 13<br>D16S539: 13,14<br>D18S51: 15,17,18 (DepMap); 15,18 (PubMed=25877200)<br>D19S433: 11,13,14<br>D21S11: 29,30,31<br>FGA: 21,26<br>Penta D: 10<br>Penta E: 6,8<br>TH01: 7,8<br>TPOX: 9<br>vWA: 18,20<br>**Run an STR similarity search on this cell line**

Note: The STR online match analysis of the test cell against EXPASY database, showing cell number (Cell No.) and cell name.

3. Authentication

☐ The submitted sample profile is human, but not a match for any profile in the DSMZ STR database.

☑ The submitted profile is an exact match for the following human cell line(s) in the EXPASY STR database (8 core loci plus Amelogenin) **C33A** .

☐ The submitted profile is similar to the following DSMZ human cell line: /.

Add: Room 502, NO.2 Zhongxing Creative Park, Lane 1015, LongtengRoad, Songjiang District, Shanghai/ Tel:+86-021-33559493
E-mail: market@biowing.com

- **Note:** A cell line can be considered to be authenticated when 80% (exact match) of the alleles in its STR profile match profiles from tissue or other cell line samples from that donor or from database. Cell lines with between a 55% to 80% (similar) match require further profiling for investigation of relatedness.

Add: Room 502, NO.2 Zhongxing Creative Park, Lane 1015, LongtengRoad, Songjiang District, Shanghai/ Tel:+86-021-33559493
E-mail: market@biowing.com

Appendix:

1. Genotyping Strategy and Site Distribution

Table S1. Experimental Strategy and Sites

	Strategy 1	Strategy 2	Strategy 3	Strategy 4
1	D3S1358	D8S1179	D19S433	AMEL
2	VWA	D21S11	TH01	D1S1656
3	D7S820	D16S539	D13S317	D5S818
4	CSF1PO	D2S1338	TPOX	D12S391
5	PENTAE	PENTAD	D18S51	FGA
6			D6S1043	

The allele match algorithm compares the 8 core loci plus amelogenin only, even though alleles from all lociwill be reported when available.

2. DSMZ tools was used to carry on the cell line comparison, which contains 2455 cell lines STR data from ATCC, DSMZ, JCRB ,ECACC， GNE and RIKEN databases. If the cell isnot included in the above cell library, users need to compared with other databases.

Technician: Jianan Zhang

Checked by: Ning Qian

Issued by: Yang Bai

Issue date: 12/24/2021

Add: Room 502, NO.2 Zhongxing Creative Park, Lane 1015, LongtengRoad, Songjiang District, Shanghai/ Tel:+86-021-33559493
E-mail: market@biowing.com
