# Supplementary material for: PDHB-AS suppresses cervical cancer progression and cisplatin resistance via inhibition on Wnt/β-catenin pathway
Source: Cell Death Dis. 2023 Feb 7;14(2):90. doi: 10.1038/s41419-022-05547-5 (PMC9905568; doi:10.1038/s41419-022-05547-5)
Supplement: Supplementary file 5 — HEK293T [file 41419_2022_5547_MOESM5_ESM.pdf]

Cell Line Authentication Service

STR Profiling Report

Sample From: Guangdong Provincial People's Hospital

Sample Type: Cell Line

Testing Method: STR Genotyping

Report Time: 12/24/2021

Add: Room 502, NO.2 Zhongxing Creative Park, Lane 1015, LongtengRoad, Songjiang District, Shanghai/ Tel:+86-021-33559493
E-mail: market@biowing.com

COMPANY STATEMENT

1. THIS REPORT IS ONLY RESPONSIBLE FOR THE SAMPLES ANALYZED.
2. THE TESTING RESULTS AND THE ORGANIZATION NAME WILL NOT BE USED FOR ADVERTISEMENT, COMMERCIAL EXHIBITIONS, COMMERCIAL PERFORMANCE AND OTHER COMMERCIAL ACTIVITIES.
3. OBJECTIONS SHOULD BE RAISED WITHIN FIFTEEN DAYS AFTER THE RECEIPT OF THIS REPORT.
4. THE PAPER REPORT WITH CONTENT ALTERING, ADDING OR WITHOUT THE STAMPED SEAL OF THE COMPANY ARE INVALID.

Testing Company: Shanghai Chenying Biotechnology Co. Ltd

Address: Room 502, NO.2 Zhongxing Creative Park, Lane 1015, LongtengRoad, Songjiang District, Shanghai

Tel: +86-021-33559493

Contact: Wenyao Zhang

E-mail: market@biowing.com

Cell Line Authentication – STR Profiling Report

Sample code

Table 1. Sample Code

Customer's code	Company Code
HEK293T	20211224-01

Sample Number:1

Sample Type: Cell line

Testing Type: STR

Testing Method:

DNA was extracted by a commercial kit from CORNING (AP-EMN-BL-GDNA-250G). The twenty STRs including Amelogenin locus were amplified by six multiplex PCR and separated on ABI 3730XL Genetic Analyzer. The signals were then analyzed by the software GeneMapper.

Data Interpretation:

Cell lines were authenticated using Short Tandem Repeat (STR) analysis asdescribed in 2012 in ANSI Standard (ASN-0002) by the ATCC Standards Development Organization (SDO) and in Capes-Davis et al., Match criteria for human cell line authentication: Where do we draw the line? Int J Cancer.2013;132(11):2510-9.

Add: Room 502, NO.2 Zhongxing Creative Park, Lane 1015, LongtengRoad, Songjiang District, Shanghai/ Tel:+86-021-33559493
E-mail: market@biowing.com

Test Results

1. STR profile

Table 2. STR and Amelogenin Genotyping Results of Cell line.

Loci	Sample information			Cell Bank information		
	Sample name: HEK293T			Cell line name: HEK293T		
	Allele1	Allele2	Allele3	Allele1	Allele2	Allele3
D5S818	8	9		8	9	
D13S317	12	12		12	12	
D7S820	11	11		11	11	
D16S539	9	13		9	13	
VWA	16	19		16	19	
TH01	7	9.3		7	9.3	
AMEL	X	X		X	X	
TPOX	11	11		11	11	
CSF1PO	11	12		11	12	
FGA	23	23				
Penta E	7	15				
Penta D	9	10				
D21S11	28	30.2				
D18S51	17	18				
D8S1179	12	14				
D3S1358	15	16	17			
D19S433	18	18				
D2S1338	19	19				

Add: Room 502, NO.2 Zhongxing Creative Park, Lane 1015, LongtengRoad, Songjiang District, Shanghai/ Tel:+86-021-33559493
E-mail: market@biowing.com

2. database annotation

Figure 1. STR matching analysis

**Cell line name**	HEK293T
**Synonyms**	Hek293T; HEK-293T; HEK 293T; HEK-293-T; HEK 293 T; 293-T; 293 T; 293T; Human Embryonic Kidney 293T; 293tsA1609neo
**Accession**	CVCL_0063
**Resource Identification Initiative**	To cite this cell line use: HEK293T (RRID:CVCL_0063)
**Comments**	Part of: ENCODE project common cell types; tier 3.<br>Part of: MD Anderson Cell Lines Project.<br>Doubling time: ~24-30 hours (DSMZ).<br>Transfected with: UniProtKB; P00552; Transposon Tn5 neo.<br>Transformant: NCBI_TaxID; 28285; Adenovirus 5.<br>Transformant: NCBI_TaxID; 1891767; Simian virus 40 (SV40) [tsA].<br>Omics: Deep proteome analysis.<br>Omics: Genome sequenced.<br>Omics: miRNA expression profiling.<br>Omics: Protein expression by reverse-phase protein arrays.<br>Misspelling: HECK293T; Occasionally.<br>Misspelling: HEK239T; Occasionally.
**Species of origin**	Homo sapiens (Human) (NCBI Taxonomy: 9606)
**Sex of cell**	Female
**Age at sampling**	Fetus
**Category**	Transformed cell line
**STR profile**	**Source(s):** ATCC; CCRID; DSMZ; ECACC; RCB<br><br>**Markers:**<br>Amelogenin: X<br>CSF1PO: 11,12<br>D2S1338: 19<br>D3S1358: 15,16,17<br>D5S818: 8,9<br>D7S820: 11<br>D8S1179: 12,14<br>D13S317: 12 (ECACC); 12,14 (ATCC; CCRID; DSMZ; RCB)<br>D16S539: 9,13<br>D18S51: 17,18<br>D19S433: 18<br>D21S11: 28,30.2<br>FGA: 23<br>Penta D: 9,10<br>Penta E: 7,15<br>TH01: 7,9.3<br>TPOX: 11<br>vWA: 16,19<br><br>**Run an STR similarity search on this cell line**

Note: The STR online match analysis of the test cell against EXPASY database, showing cell number (Cell No.) and cell name.

3. Authentication

☐ The submitted sample profile is human, but not a match for any profile in the DSMZ STR database.

☑ The submitted profile is an exact match for the following human cell line(s) in the EXPASY STR database (8 core loci plus Amelogenin) **HEK293T** .

☐ The submitted profile is similar to the following DSMZ human cell line: /.

- **Note:** A cell line can be considered to be authenticated when 80% (exact match) of the alleles in its STR profile match profiles from tissue or other cell line samples from that donor or from database. Cell lines with between a 55% to 80% (similar) match require further profiling for investigation of relatedness.

Add: Room 502, NO.2 Zhongxing Creative Park, Lane 1015, LongtengRoad, Songjiang District, Shanghai/ Tel:+86-021-33559493
E-mail: market@biowing.com

Appendix:

1. Genotyping Strategy and Site Distribution

Table S1. Experimental Strategy and Sites

	Strategy 1	Strategy 2	Strategy 3	Strategy 4
1	D3S1358	D8S1179	D19S433	AMEL
2	VWA	D21S11	TH01	D1S1656
3	D7S820	D16S539	D13S317	D5S818
4	CSF1PO	D2S1338	TPOX	D12S391
5	PENTAE	PENTAD	D18S51	FGA
6			D6S1043	

The allele match algorithm compares the 8 core loci plus amelogenin only, even though alleles from all lociwill be reported when available.

2. DSMZ tools was used to carry on the cell line comparison, which contains 2455 cell lines STR data from ATCC, DSMZ, JCRB ,ECACC， GNE and RIKEN databases. If the cell is not included in the above cell library, users need to compared with other databases.

Technician: Jianan Zhang

Checked by: Ning Qian

Issued by: Yang Bai

Issue date: 12/24/2021

Add: Room 502, NO.2 Zhongxing Creative Park, Lane 1015, LongtengRoad, Songjiang District, Shanghai/ Tel:+86-021-33559493
E-mail: market@biowing.com
