# Supplementary material for: PDHB-AS suppresses cervical cancer progression and cisplatin resistance via inhibition on Wnt/β-catenin pathway
Source: Cell Death Dis. 2023 Feb 7;14(2):90. doi: 10.1038/s41419-022-05547-5 (PMC9905568; doi:10.1038/s41419-022-05547-5)
Supplement: Supplementary file 6 — HeLa [file 41419_2022_5547_MOESM6_ESM.pdf]

Cell Line Authentication Service

STR Profiling Report

Sample From: Guangdong Provincial People's Hospital
Sample Type: Cell Line
Testing Method: STR Genotyping
Report Time: 12/24/2021

Add: Room 502, NO.2 Zhongxing Creative Park, Lane 1015, LongtengRoad, Songjiang District, Shanghai/ Tel:+86-021-33559493
E-mail: market@biowing.com

COMPANY STATEMENT

1. THIS REPORT IS ONLY RESPONSIBLE FOR THE SAMPLES ANALYZED.
2. THE TESTING RESULTS AND THE ORGANIZATION NAME WILL NOT BE USED FOR ADVERTISEMENT, COMMERCIAL EXHIBITIONS, COMMERCIAL PERFORMANCE AND OTHER COMMERCIAL ACTIVITIES.
3. OBJECTIONS SHOULD BE RAISED WITHIN FIFTEEN DAYS AFTER THE RECEIPT OF THIS REPORT.
4. THE PAPER REPORT WITH CONTENT ALTERING, ADDING OR WITHOUT THE STAMPED SEAL OF THE COMPANY ARE INVALID.

Testing Company: Shanghai Chenying Biotechnology Co. Ltd

Address: Room 502, NO.2 Zhongxing Creative Park, Lane 1015, LongtengRoad, Songjiang District, Shanghai

Tel: +86-021-33559493

Contact: Wenyao Zhang

E-mail: market@biowing.com

Cell Line Authentication – STR Profiling Report

Sample code

Table 1. Sample Code

Customer's code	Company Code
HeLa	20211224-01

Sample Number:1

Sample Type: Cell line

Testing Type: STR

Testing Method:

DNA was extracted by a commercial kit from CORNING (AP-EMN-BL-GDNA-250G). The twenty STRs including Amelogenin locus were amplified by six multiplex PCR and separated on ABI 3730XL Genetic Analyzer. The signals were then analyzed by the software GeneMapper.

Data Interpretation:

Cell lines were authenticated using Short Tandem Repeat (STR) analysis asdescribed in 2012 in ANSI Standard (ASN-0002) by the ATCC Standards Development Organization (SDO) and in Capes-Davis et al., Match criteria for human cell line authentication: Where do we draw the line? Int J Cancer.2013;132(11):2510-9.

Test Results

1. STR profile

Table 2. STR and Amelogenin Genotyping Results of Cell line.

Loci	Sample information			Cell Bank information		
	Sample name：HeLa			Cell line name：HeLa		
	**Allele1**	**Allele2**	**Allele3**	**Allele1**	**Allele2**	**Allele3**
D5S818	11	12		11	12	
D13S317	12	13.3		12	13.3	
D7S820	8	12		8	12	
D16S539	9	10		9	10	
VWA	16	18		16	18	
TH01	7			7		
AMEL	X	X		X	X	
TPOX	8	12		8	12	
CSF1PO	9	10		9	10	
FGA	18	21				
Penta E	7	17				
Penta D	8	15				
D21S11	27	28				
D18S51	16					
D8S1179	12	13				
D3S1358	15	18				
D19S433	13	14				
D2S1338	17					
D1S1656	12	15				
D2S441	10	11				
D10S1248	13	15				

Add: Room 502, NO.2 Zhongxing Creative Park, Lane 1015, LongtengRoad, Songjiang District, Shanghai/ Tel:+86-021-33559493
E-mail: market@biowing.com

D12S391	20	25	
D22S1045	16	17	

Add: Room 502, NO.2 Zhongxing Creative Park, Lane 1015, LongtengRoad, Songjiang District, Shanghai/ Tel:+86-021-33559493
E-mail: market@biowing.com

2. database annotation

Figure 1. STR matching analysis

**Cell line name**	HeLa
**Synonyms**	HELA; Hela; He La; He-La; HeLa-CCL2; Henrietta Lacks cells; Helacyton gartleri
**Accession**	CVCL_0030
**Resource Identification Initiative**	To cite this cell line use: HeLa (RRID:CVCL_0030)
**Comments**	Group: Space-flown cell line (cellonaut).<br>Part of: Cancer Dependency Map project (DepMap) (includes Cancer Cell Line Encyclopedia - CCLE).<br>Part of: COSMIC cell lines project.<br>Part of: MD Anderson Cell Lines Project.<br>Part of: Naval Biosciences Laboratory (NBL) collection (transferred to ATCC in 1982).<br>Population: African American.<br>Characteristics: HeLa has 5 five HPV18 integration sites: three on normal chromosomes 8 at 8q24 and two on derivative chromosomes, der(5)t(5;22;8)(q11;q11q13;q24) and der(22)t(8;22)(q24;q13).<br>Characteristics: Not susceptible to SARS coronavirus 2 (SARS-CoV-2) infection (COVID-19) (PubMed=33389257).<br>Doubling time: 1.3 days (PubMed=29156801); ~48 hours (DSMZ).<br>Microsatellite instability: Stable (MSS) (PubMed=12661003; Sanger).<br>Transformant: NCBI_TaxID; 333761; Human papillomavirus type 18 (HPV18).<br>Omics: Cell surface proteome.<br>Omics: CNV analysis.<br>Omics: Deep antibody staining analysis.<br>Omics: Deep exome analysis.<br>Omics: Deep membrane proteome analysis.<br>Omics: Deep phosphoproteome analysis.<br>Omics: Deep proteome analysis.<br>Omics: Deep quantitative proteome analysis.<br>Omics: Deep RNAseq analysis.<br>Omics: DNA methylation analysis.<br>Omics: Genome sequenced.<br>Omics: Glycoproteome analysis by proteomics.<br>Omics: Myristoylated proteins analysis by proteomics.<br>Omics: Protein expression by reverse-phase protein arrays.<br>Omics: scRNAseq analysis.<br>Omics: SNP array analysis.<br>Omics: Transcriptome analysis.<br>Omics: Virome analysis using proteomics.<br>Anecdotal: The HeLa cell line which was established in February 1951 is the oldest human immortal cell line.<br>Anecdotal: The fascinating story of the HeLa cell line and of Henrietta Lacks from whom these cells originate are described in the book of Rebecca Skloot (CelloPub=CLPUB00377).<br>Anecdotal: The HeLa cell line and its story inspired Australian artist Cynthia Verspaget to embark in 2003 on a artistic project 'The Anarchy Cell Line' (TAnCL) where she mixed her blood with HeLa cells. This work later spawned a PhD thesis (CelloPub=CLPUB00376) where among other things she makes the observation that two main taxonomical distinctions present in the zombie, living/dead and human/nonhuman, are also present in the HeLa cell line.<br>Anecdotal: Was flown since the 1960s on at least ten different space missions: Korabl-Sputnik-2, Vostok-1, Vostok-4, Vostok-5 and Vostok-6, Voshkod 1 and Zond-5, Discoverer XVIII, Progress M-35/Mir and Shuttle STS-89.<br>Miscellaneous: HeLa is the most frequent contributor to cell lines contamination.<br>Derived from sampling site: Uterus; cervix.
**Species of origin**	Homo sapiens (Human) (NCBI Taxonomy: 9606)
**Sex of cell**	Female
**Age at sampling**	30Y6M
**Category**	Cancer cell line
**STR profile**	**Source(s):** AddexBio; ATCC; CCRID; CLS; Cosmic-CLP; DSMZ; ECACC; Genomics_Center_BCF_Technion; JCRB; KCLB; PubMed=11416159; PubMed=25877200; PubMed=29156801; RCB; TKG<br><br>**Markers:**

Marker	Alleles
Amelogenin	X
CSF1PO	9,10
D1S1656	12,15
D2S441	10,11
D2S1338	17
D3S1358	15,18
D5S818	11,12
D7S820	8,12
D8S1179	12,13
D10S1248	13,15
D12S391	20,25
D13S317	12 (Cosmic-CLP; RCB)<br>12,13.3 (AddexBio; ATCC; CCRID; DSMZ; Genomics_Center_BCF_Technion; JCRB; KCLB; PubMed=25877200; PubMed=29156801; TKG)<br>12,14 (ECACC)<br>13,13.3 (CLS)
D16S539	9,10
D18S51	16
D19S433	13,14
D21S11	27 (CLS)<br>27,28 (CCRID; DSMZ; Genomics_Center_BCF_Technion; PubMed=25877200)
D22S1045	16,17
FGA	18,21
Penta D	8 (CLS)<br>8,15 (CCRID; DSMZ; Genomics_Center_BCF_Technion; PubMed=25877200)
Penta E	7,17
TH01	7
TPOX	8,12
vWA	16,18

Run an STR similarity search on this cell line

Add: Room 502, NO.2 Zhongxing Creative Park, Lane 1015, LongtengRoad, Songjiang District, Shanghai/ Tel:+86-021-33559493
E-mail: market@biowing.com

Note: The STR online match analysis of the test cell against EXPASY database, showing cell number (Cell No.) and cell name.

3. Authentication

☐ The submitted sample profile is human, but not a match for any profile in the DSMZ STR database.

☑ The submitted profile is an exact match for the following human cell line(s) in the EXPASY STR database (8 core loci plus Amelogenin) **HeLa** .

☐ The submitted profile is similar to the following DSMZ human cell line: /.

- **Note:** A cell line can be considered to be authenticated when 80% (exact match) of the alleles in its STR profile match profiles from tissue or other cell line samples from that donor or from database. Cell lines with between a 55% to 80% (similar) match require further profiling for investigation of relatedness.

Add: Room 502, NO.2 Zhongxing Creative Park, Lane 1015, LongtengRoad, Songjiang District, Shanghai/ Tel:+86-021-33559493
E-mail: market@biowing.com

Appendix:

1. Genotyping Strategy and Site Distribution

Table S1. Experimental Strategy and Sites

	Strategy 1	Strategy 2	Strategy 3	Strategy 4
1	D3S1358	D8S1179	D19S433	AMEL
2	VWA	D21S11	TH01	D1S1656
3	D7S820	D16S539	D13S317	D5S818
4	CSF1PO	D2S1338	TPOX	D12S391
5	PENTAE	PENTAD	D18S51	FGA
6			D6S1043	

The allele match algorithm compares the 8 core loci plus amelogenin only, even though alleles from all lociwill be reported when available.

2. DSMZ tools was used to carry on the cell line comparison, which contains 2455 cell lines STR data from ATCC, DSMZ, JCRB ,ECACC， GNE and RIKEN databases. If the cell is not included in the above cell library, users need to compared with other databases.

Technician: Jianan Zhang

Checked by: Ning Qian

Issued by: Yang Bai

Issue date: 12/24/2021

Add: Room 502, NO.2 Zhongxing Creative Park, Lane 1015, LongtengRoad, Songjiang District, Shanghai/ Tel:+86-021-33559493
E-mail: market@biowing.com
