# Supplementary material for: PDHB-AS suppresses cervical cancer progression and cisplatin resistance via inhibition on Wnt/β-catenin pathway
Source: Cell Death Dis. 2023 Feb 7;14(2):90. doi: 10.1038/s41419-022-05547-5 (PMC9905568; doi:10.1038/s41419-022-05547-5)
Supplement: Supplementary file 7 — ME-180 [file 41419_2022_5547_MOESM7_ESM.pdf]

# Cell Line Authentication Service

## STR Profiling Report

**Sample From:** Guangdong Provincial People's Hospital

**Sample Type:** Cell Line

**Testing Method:** STR Genotyping

**Report Time:** 12/24/2021


Add: Room 502, NO.2 Zhongxing Creative Park, Lane 1015, LongtengRoad, Songjiang District, Shanghai/ Tel:+86-021-33559493
E-mail: market@biowing.com

# COMPANY STATEMENT

1. THIS REPORT IS ONLY RESPONSIBLE FOR THE SAMPLES ANALYZED.
2. THE TESTING RESULTS AND THE ORGANIZATION NAME WILL NOT BE USED FOR ADVERTISEMENT, COMMERCIAL EXHIBITIONS, COMMERCIAL PERFORMANCE AND OTHER COMMERCIAL ACTIVITIES.
3. OBJECTIONS SHOULD BE RAISED WITHIN FIFTEEN DAYS AFTER THE RECEIPT OF THIS REPORT.
4. THE PAPER REPORT WITH CONTENT ALTERING, ADDING OR WITHOUT THE STAMPED SEAL OF THE COMPANY ARE INVALID.

**Testing Company:** Shanghai Chenying Biotechnology Co. Ltd

**Address:** Room 502, NO.2 Zhongxing Creative Park, Lane 1015, LongtengRoad, Songjiang District, Shanghai

**Tel:** +86-021-33559493

**Contact:** Wenyao Zhang

**E-mail:** market@biowing.com

# Cell Line Authentication – STR Profiling Report

## Sample code

Table 1. Sample Code

| Customer's code | Company Code |
|---|---|
| ME-180 | 20211224-01 |

**Sample Number**:1

**Sample Type**: Cell line

**Testing Type**: STR

**Testing Method**:

DNA was extracted by a commercial kit from CORNING (AP-EMN-BL-GDNA-250G). The twenty STRs including Amelogenin locus were amplified by six multiplex PCR and separated on ABI 3730XL Genetic Analyzer. The signals were then analyzed by the software GeneMapper.

## Data Interpretation:

Cell lines were authenticated using Short Tandem Repeat (STR) analysis asdescribed in 2012 in ANSI Standard (ASN-0002) by the ATCC Standards Development Organization (SDO) and in Capes-Davis et al., Match criteria for human cell line authentication: Where do we draw the line? Int J Cancer.2013;132(11):2510-9.


Add: Room 502, NO.2 Zhongxing Creative Park, Lane 1015, LongtengRoad, Songjiang District, Shanghai/ Tel:+86-021-33559493
E-mail: market@biowing.com

# Test Results

## 1. STR profile

Table 2. STR and Amelogenin Genotyping Results of Cell line.

| Loci | **Sample information** | | | **Cell Bank information** | | |
|---|---|---|---|---|---|---|
| | Sample name：ME-180 | | | Cell line name：ME-180 | | |
| | **Allele1** | **Allele2** | **Allele3** | **Allele1** | **Allele2** | **Allele3** |
| D5S818 | 12 | | | 12 | | |
| D13S317 | 11 | 13 | | 11 | 13 | |
| D7S820 | 9 | 10 | | 9 | 10 | |
| D16S539 | 12 | 13 | | 12 | 13 | |
| VWA | 15 | 17 | | 15 | 17 | |
| TH01 | 8 | 9.3 | | 8 | 9.3 | |
| AMEL | X | | | X | | |
| TPOX | 8 | 10 | | 8 | 10 | |
| CSF1PO | 11 | | | 11 | | |
| FGA | 23 | | | | | |
| Penta E | 12 | 14 | | | | |
| Penta D | 9 | 14 | | | | |
| D21S11 | 30 | 31 | | | | |
| D18S51 | 12 | | | | | |
| D8S1179 | 14 | | | | | |
| D3S1358 | 16 | | | | | |
| D19S433 | 13 | 15.2 | | | | |
| D2S1338 | 18 | | | | | |


Add: Room 502, NO.2 Zhongxing Creative Park, Lane 1015, LongtengRoad, Songjiang District, Shanghai/ Tel:+86-021-33559493
E-mail: market@biowing.com

## 2. database annotation

Figure 1. STR matching analysis

| | |
|---|---|
| **Cell line name** | ME-180 |
| **Synonyms** | Me-180; ME 180; ME180 |
| **Accession** | CVCL_1401 |
| **Resource Identification Initiative** | To cite this cell line use: ME-180 (RRID:CVCL_1401) |
| **Comments** | Part of: Cancer Dependency Map project (DepMap) (includes Cancer Cell Line Encyclopedia - CCLE).<br>Part of: COSMIC cell lines project.<br>Part of: Naval Biosciences Laboratory (NBL) collection (transferred to ATCC in 1982).<br>Characteristics: Contains a complete HPV68 genome.<br>Doubling time: 1.5 days (PubMed=29156801).<br>Microsatellite instability: Stable (MSS) (Sanger).<br>Transformant: NCBI_TaxID; 45240; Human papillomavirus type 68 (HPV68).<br>Omics: Deep exome analysis.<br>Omics: Deep proteome analysis.<br>Omics: Deep RNAseq analysis.<br>Omics: DNA methylation analysis.<br>Omics: Protein expression by reverse-phase protein arrays.<br>Omics: SNP array analysis.<br>Omics: Transcriptome analysis. |
| **Species of origin** | Homo sapiens (Human) (NCBI Taxonomy: 9606) |
| **Sex of cell** | Female |
| **Age at sampling** | 66Y |
| **Category** | Cancer cell line |
| **STR profile** | **Source(s):** ATCC; CCRID; Cosmic-CLP; JCRB; KCLB; PubMed=22710073; PubMed=25877200; PubMed=29156801; RCB; TKG<br><br>**Markers:** |

| Marker | Alleles |
|---|---|
| Amelogenin | X |
| CSF1PO | 11 |
| D2S1338 | 18 |
| D3S1358 | 16 |
| D5S818 | 12 |
| D7S820 | 9,10 |
| D8S1179 | 14 |
| D13S317 | 11,12 (PubMed=22710073)<br>11,13 (ATCC; CCRID; Cosmic-CLP; JCRB; KCLB; PubMed=25877200; PubMed=29156801; RCB; TKG) |
| D16S539 | 12,13 |
| D18S51 | 12 |
| D19S433 | 13,15.2 |
| D21S11 | 30,31 |
| FGA | 23 |
| Penta D | 9,14 |
| Penta E | 12,14 |
| TH01 | 8 (RCB)<br>8,9.3 (ATCC; CCRID; Cosmic-CLP; JCRB; KCLB; PubMed=22710073; PubMed=25877200; PubMed=29156801; TKG) |
| TPOX | 8,10 |
| vWA | 15,16,17 (JCRB)<br>15,17 (ATCC; CCRID; Cosmic-CLP; KCLB; PubMed=22710073; PubMed=25877200; PubMed=29156801; RCB; TKG) |

**Run an STR similarity search on this cell line**

**Note:** The STR online match analysis of the test cell against EXPASY database, showing cell number (Cell No.) and cell name.

## 3. Authentication

☐ The submitted sample profile is human, but not a match for any profile in the DSMZ STR database.

☑ The submitted profile is an exact match for the following human cell line(s) in the EXPASY STR database (8 core loci plus Amelogenin) **ME-180** .

☐ The submitted profile is similar to the following DSMZ human cell line: /.

- **Note:** A cell line can be considered to be authenticated when 80% (exact match) of the


Add: Room 502, NO.2 Zhongxing Creative Park, Lane 1015, LongtengRoad, Songjiang District, Shanghai/ Tel:+86-021-33559493
E-mail: market@biowing.com

alleles in its STR profile match profiles from tissue or other cell line samples from that donor or from database. Cell lines with between a 55% to 80% (similar) match require further profiling for investigation of relatedness.


Add: Room 502, NO.2 Zhongxing Creative Park, Lane 1015, LongtengRoad, Songjiang District, Shanghai/ Tel:+86-021-33559493
E-mail: market@biowing.com

# Appendix:

1. Genotyping Strategy and Site Distribution

Table S1. Experimental Strategy and Sites

| | Strategy 1 | Strategy 2 | Strategy 3 | Strategy 4 |
|---|---|---|---|---|
| 1 | D3S1358 | D8S1179 | D19S433 | AMEL |
| 2 | VWA | D21S11 | TH01 | D1S1656 |
| 3 | D7S820 | D16S539 | D13S317 | D5S818 |
| 4 | CSF1PO | D2S1338 | TPOX | D12S391 |
| 5 | PENTAE | PENTAD | D18S51 | FGA |
| 6 | | | D6S1043 | |

*The allele match algorithm compares the 8 core loci plus amelogenin only, even though alleles from all lociwill be reported when available.*

2. DSMZ tools was used to carry on the cell line comparison, which contains 2455 cell lines STR data from ATCC, DSMZ, JCRB ,ECACC， GNE and RIKEN databases. If the cell is not included in the above cell library, users need to compared with other databases.

**Technician:** Jianan Zhang

**Checked by:** Ning Qian

**Issued by**: Yang Bai

**Issue date:** 12/24/2021


Add: Room 502, NO.2 Zhongxing Creative Park, Lane 1015, LongtengRoad, Songjiang District, Shanghai/ Tel:+86-021-33559493
E-mail: market@biowing.com
