# Supplementary figures and images for: PDHB-AS suppresses cervical cancer progression and cisplatin resistance via inhibition on Wnt/β-catenin pathway
Source: Cell Death Dis. 2023 Feb 7;14(2):90. doi: 10.1038/s41419-022-05547-5 (PMC9905568; doi:10.1038/s41419-022-05547-5)

1G

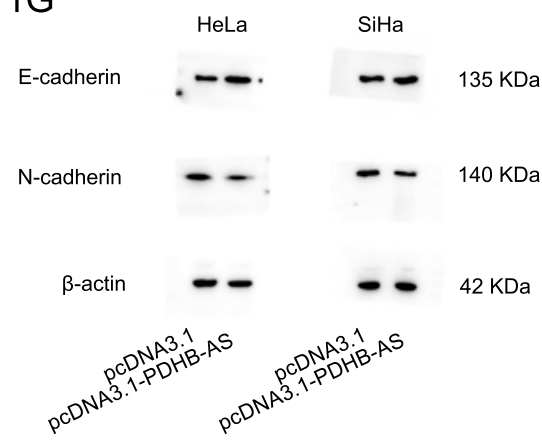

2C

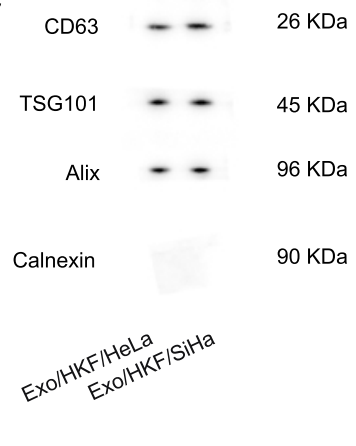

2G

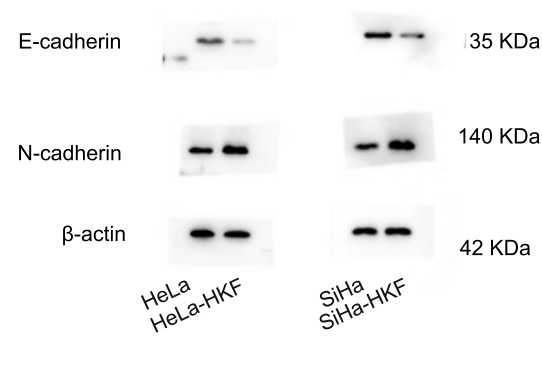

3D

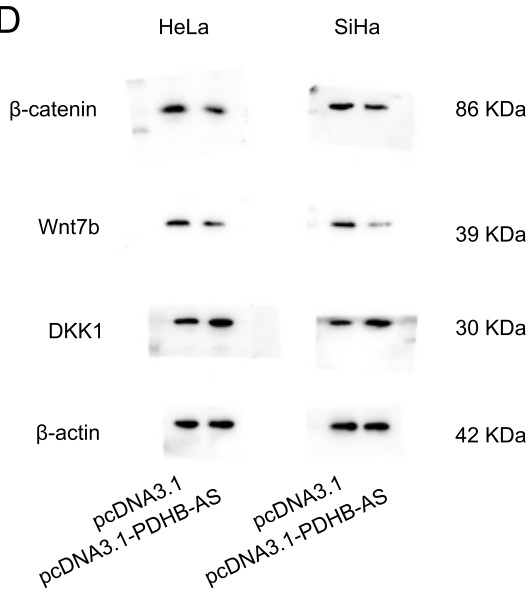

3E

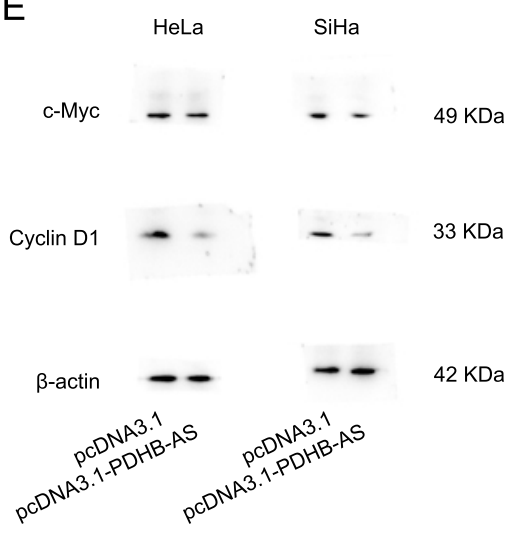

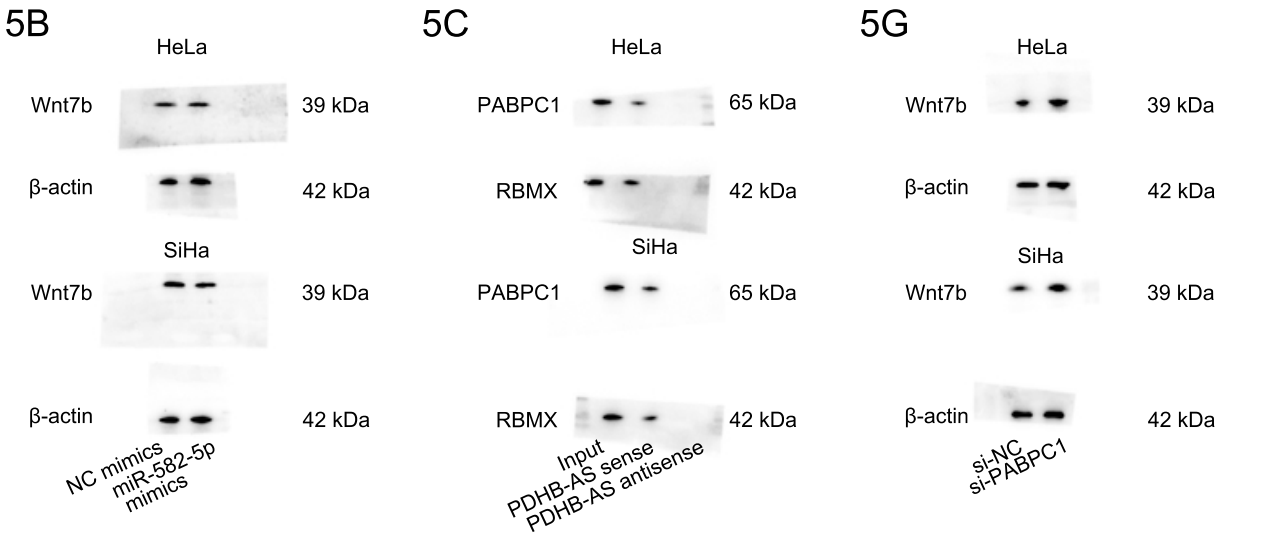

Supplement: Supplementary file 10 — Original Data File [file 41419_2022_5547_MOESM10_ESM.pdf]
