# Supplementary material for: PDHB-AS suppresses cervical cancer progression and cisplatin resistance via inhibition on Wnt/β-catenin pathway
Source: Cell Death Dis. 2023 Feb 7;14(2):90. doi: 10.1038/s41419-022-05547-5 (PMC9905568; doi:10.1038/s41419-022-05547-5)
Supplement: Supplementary file 11 — Authorship change file [file 41419_2022_5547_MOESM11_ESM.pdf]

DECLARATION OF CONTRIBUTIONS TO ARTICLE

ADMC

Manuscript Number:	Journal Name:	
	Cell Death & Disease	(the 'Journal')

Proposed Title of the Contribution:

The author's contribution	(the 'Contribution)

Author(s):

Chi Chi, Wenjie Hou, Yi Zhang, Jie Chen, Zongji Shen, Min Li, Youguo Chen	(the 'Authors')

For all *CDDis* articles, each person named as an author in the published version must be able to show he or she has contributed substantially to the article.

Authorship credit should be based on 1) substantial contributions to conception and design, acquisition of data, or analysis and interpretation of data; 2) drafting the article or revising it critically for important intellectual content; and 3) final approval of the version to be published. Authors should meet conditions 1, 2 and 3.

Any person who cannot be shown to have made a substantial contribution to the article cannot be listed as an author in the final version. The name of any person who is deemed to have made a minor contribution can, however, appear in the Acknowledgments section of the article.

Please complete the table below to indicate the contributions of all named authors to the manuscript.

Author Full Name:	Specification of Contribution to the Manuscript:
Chi Chi	wrote the paper
Wenjie Hou	administrated the whole study, approved the final manuscript
Yi Zhang	designed the experiment
Jie Chen	did the statistical analysis
Zongji Shen	contributed to the resources
Min Li	designed the experiment
Youguo Chen	did the statistical analysis

Please complete the table below to indicate the contributions of all named authors to the figures.

Figure 1,2,3:

Yi Zhang

Figure 4,5,6,7,8:

Min Li

Figure S1,S2,S3:

Figure S4:

Signed for and on behalf of the Author(s):	Print Name:	Date:
Chi Chi	Chi Chi	2020.12.29
